# Refinement of organic crystal structures with multipolar electron scattering factors

**DOI:** 10.1107/S2053273319015304

**Published:** 2020-01-01

**Authors:** Barbara Gruza, Michał Leszek Chodkiewicz, Joanna Krzeszczakowska, Paulina Maria Dominiak

**Affiliations:** a Biological and Chemical Research Centre, Department of Chemistry, University of Warsaw, ul. Żwirki i Wigury 101, Warsaw, 02-089, Poland

**Keywords:** electron crystallography, electron microscopy, electron diffraction, aspherical scattering factors, structure refinement, transferable aspherical atom model (TAAM), quantum crystallography, micro-electron diffraction, cryo-electron microscopy

## Abstract

A revolution in resolution is occurring now in electron microscopy arising from the development of methods for imaging single particles at cryogenic temperatures and obtaining electron diffraction data from nanocrystals of small organic molecules or macromolecules. Near-atomic or even atomic resolution of molecular structures can be achieved. The basis of these methods is the scattering of an electron beam due to the electrostatic potential of the sample. To analyse these high-quality experimental data, it is necessary to use appropriate atomic scattering factors. The independent atom model (IAM) is commonly used although various more advanced models, already known from X-ray diffraction, can also be applied to enhance the analysis. In this study a comparison is presented of IAM and TAAM (transferable aspherical atom model), the latter with the parameters of the Hansen–Coppens multipole model transferred from the University at Buffalo Databank (UBDB). By this method, TAAM takes into account the fact that atoms in molecules are partially charged and are not spherical. Structure refinements were performed on a carbamazepine crystal using electron structure-factor amplitudes determined experimentally [Jones *et al.* (2018 ▸). *ACS Cent. Sci.* **4**, 1587–1592] or modelled with theoretical quantum-mechanical methods. The results show the possibilities and limitations of the TAAM method when applied to electron diffraction. Among others, the method clearly improves model fitting statistics, when compared with IAM, and allows for reliable refinement of atomic thermal parameters. The improvements are more pronounced with poorer-resolution diffraction data.

## Abstract 2

Precomputed multipolar electron scattering factors are employed for electron crystallography. Because this takes into account the fact that atoms are partially charged and aspherical, model fitting statistics and atomic thermal parameters are visibly improved.

## 1. Introduction   

Until recently, the only techniques that routinely yielded atomic and near-atomic resolution structures of molecules were X-ray crystallography and nuclear magnetic resonance (NMR) spectroscopy. However, new technological and computational developments for transmission electron microscopes have made electron scattering based techniques legitimate candidates for routine 3D structure determinations at atomic resolution. Nowadays, single-particle cryo-electron microscopy (cryo-EM) can be used to determine near-atomic resolution structures of macromolecules that are either reluctant to crystallize or are difficult to crystallize in specific functional states. Electron diffraction (ED), on the other hand, is currently the method of choice to study crystal structures and properties of nano-sized materials at atomic resolutions. These include materials for which it is difficult to obtain crystals of a size suitable for X-ray analysis, like pharma­ceuticals, pigments, zeolites and macromolecules.

The race to achieve near-atomic resolution structures using cryo-EM has accelerated over the past few years (Binshtein & Ohi, 2015 ▸; Cheng *et al.*, 2015 ▸; Subramaniam *et al.*, 2016 ▸; Merk *et al.*, 2016 ▸; Cheng, 2015 ▸; Dubochet *et al.*, 2017 ▸). The field is still developing and it is likely that there will be further advances in resolution, a decrease in the minimal size requirements for studied macromolecules, automation of data collection and improvements in ease of use (Carroni & Saibil, 2016 ▸). The same exciting developments will also allow electron crystallographers to determine crystal structures from micro- and nanocrystals with increasing accuracy and astonishing levels of detail (de la Cruz *et al.*, 2017 ▸; Palatinus *et al.*, 2017 ▸). Over the past decade, electron crystallography has been developed to a level approaching X-ray crystallography in terms of both the robustness of the structure determination and the accuracy of the inferred structure models (Dudka *et al.*, 2008 ▸; Sawaya *et al.*, 2016 ▸; Jones *et al.*, 2018 ▸; Clabbers *et al.*, 2019 ▸; Gruene *et al.*, 2018 ▸; Krysiak *et al.*, 2018 ▸).

Cryo-EM and ED methods rely on elastic interactions between the electrons and the specimen, whether single-particle or crystal. In these techniques, an electron beam is scattered by the specimens’ electrostatic (Coulomb) potential generated by charges on the nucleus screened by the charge electron density. Electron scattering is much more sensitive to fine details within the electron-density distribution than X-ray scattering. This is because of ‘*the near cancellation of the electron scattering from the positively charged nucleus and the negatively charged electrons*’ (Zheng *et al.*, 2005 ▸). To properly interpret collected scattered/diffracted beams, a model of the electrostatic potential is built using precomputed electron scattering factors from the individual atoms. For the majority of structures an independent atom model (IAM) is used (Brown *et al.*, 2015 ▸; Hryc *et al.*, 2017 ▸; Petrícek *et al.*, 2014 ▸), for which electron scattering factors were computed for independent, spherically averaged, neutral atoms or ions (Cowley *et al.*, 2006 ▸).

Electron scattering is affected by local electric charges and ionization states. Yonekura and co-workers have shown (Yonekura & Maki-Yonekura, 2016 ▸) that ‘*electron scattering factors of charged atoms vary considerably over a range of spatial frequency depending on the charged state*’. Furthermore, they demonstrated the applicability of partially charged scattering factors for refinement of the atomic models against either ED data from crystals of Ca^2+^-ATPase and catalase [3.4 Å and 3.2 Å resolution, respectively (Yonekura *et al.*, 2015 ▸)] or a cryo-EM map of β-galactosidase obtained by single-particle analysis [2.2 Å resolution (Bartesaghi *et al.*, 2014 ▸)]. Yonekura & Maki-Yonekura (2016 ▸) obtained improved fitting statistics and observed smaller temperature factors for the carboxyl oxygen atoms of the Asp and Glu side chains. In fact, the effect of using improper electron scattering factors is apparent in many of the currently available 3D cryo-EM maps (Hryc *et al.*, 2017 ▸; de la Cruz *et al.*, 2017 ▸; Shi *et al.*, 2013 ▸; Nannenga *et al.*, 2014 ▸). The usage of improper electron scattering factors may also explain the problems observed in refinement of temperature factors when trying to produce physically meaningful values (Wlodawer *et al.*, 2017 ▸).

The above considerations touch on only those applications of proper partial charges to the electron scattering factors. In reality, the electron densities of atoms in molecules are not spherical. For the aspherical case, the scattering amplitudes depend strongly upon the direction of the scattering vector. Zheng and co-workers (Zheng *et al.*, 2009 ▸) tabulated angular-dependent electron scattering factors for aspherical *p* and *d* orbitals for atoms with atomic number 1 (H) to 54 (Xe). However, to employ these scattering factors, the orientation of the orbitals of each atom in the studied molecule must be known. Zhong and co-workers (Zhong *et al.*, 2002 ▸) proposed computing potentials for small molecular fragments and from these, electron scattering factors. In their proof-of-concept study they tested the usage of different atomic and molecular fragments for reproducing the molecular electrostatic potential of different conformations of *N*-acetyl­alanine methyl­amide (NAAMA). During their ED theoretical simulations, they noticed that ‘*in the resolution range 2.5–25 Å, the fairly straightforward use of single atoms in molecules reduces the calculated R factors by 5–15% over a free-atom superposition*’. Finally they stated: ‘*In addition to experimental limitations that currently affect the accuracy of F_obs_, it is likely that the large values of R factors seen in the electron crystallographic structures are also partly attributable to errors in computing F_calc_ using scattering factors for free (i.e. unbonded and neutral) atoms*’.

At this point it must be remembered that, because of strong interactions between the electrons and the crystal, for ED multiple scattering effects among exiting beams are hardly avoidable. In the presence of multiple scattering effects, the diffraction data can no longer be interpreted using a purely kinematic approximation where *I*(**h**) ∼ |*F*(**h**)|^2^. Therefore, quantitative data analysis has to be performed based on dynamic scattering calculations (Palatinus, Corrêa *et al.*, 2015 ▸; Palatinus, Petrícek & Corrêa, 2015 ▸; Palatinus *et al.*, 2017 ▸). Nevertheless, *ab initio* structure solution requires knowledge of the structural amplitudes in the kinematic approximation (Dudka *et al.*, 2008 ▸). If the crystalline sample is sufficiently thin then this will ensure that the measured data are pre­dominantly kinematic and multiple scattering effects should not severely hamper structure solution and refinement (Clabbers *et al.*, 2017 ▸; Palatinus *et al.*, 2017 ▸).

In X-ray crystallography the limitation of IAM has been widely recognized and overcome by the multipole atom model. In the multipole model an atom is represented as a finite spherical harmonic expansion of the electron density around each atomic centre (*e.g*. a pseudoatom). In a commonly used variant of the model, the so-called Hansen–Coppens multipolar model (Hansen & Coppens, 1978 ▸), the electron density of a pseudoatom is defined by
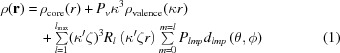


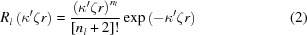
where ρ_core_(*r*) and ρ_valence_(*r*) are the spherically averaged free-atom core and the valence densities which are normalized to one electron, respectively; *R_l_*(κ′ζ*r*) is a Slater-type radial function with predefined values of ζ and *n_l_* while *d_lmp_*(θ, ϕ) (*p* = ±) is a density-normalized real spherical harmonic function. The coordinates *r*, θ, ϕ refer to a local Cartesian coordinate system centred on the atomic nucleus. The populations *P_v_* and *P_lmp_*, and the dimensionless expansion–contraction parameters κ and κ′ are parameters that are allowed to vary from one atom to another. Such a definition allows the pseudoatom electron density to be individually adjusted (by changing values of pseudoatom parameters) to account for density departures from the spherical and neutral models.

The values of pseudoatom parameters are typically almost identical for atoms in similar chemical environments (Pichon-Pesme *et al.*, 1995 ▸), *i.e.* atoms having similar local topologies of connecting chemical bonds. Therefore it was possible to build a databank of different types of pseudoatoms and to use the databank to generate transferable aspherical atom model (TAAM) parameters for any organic molecule, up to and including proteins and nucleic acids. There are three major pseudoatom databanks available at the moment: ELMAM2 (Zarychta *et al.*, 2007 ▸; Domagala & Jelsch, 2008 ▸; Domagała *et al.*, 2011 ▸, 2012 ▸), the Invariom database called GID (Dittrich *et al.*, 2006 ▸, 2013 ▸) and the University at Buffalo Pseudoatom Databank (UBDB) (Koritsanszky *et al.*, 2002 ▸; Volkov, Li *et al.*, 2004 ▸; Dominiak *et al.*, 2007 ▸; Jarzembska & Dominiak, 2012 ▸; Kumar *et al.*, 2019 ▸). They differ principally in the sources used to define the pseudoatom parameters and in the method by which atom types are defined.

Since 2005 we have been involved in the development of the UBDB (Kumar *et al.*, 2019 ▸; Jarzembska & Dominiak, 2012 ▸; Dominiak *et al.*, 2007 ▸). Currently the UBDB contains atom types occurring in proteins, nucleic acids and many organic molecules. In the UBDB, the definitions of each atom type result from averaging of density parameters (*P_v_*, *P_lmp_*, κ and κ′) for the entire family of pseudo­atoms which are considered to be chemically equivalent. The databank is derived from theoretical electron densities generated by fitting the pseudo­atom parameters in Fourier space to molecular electron densities determined by quantum-mechanical methods for a series of small molecules. The electron densities are obtained from B3LYP/6-31G** single-point calculations on the basis of experimental geometries taken from the Cambridge Structural Database (CSD; Groom *et al.*, 2016 ▸). For different atom types both the first and second neighbour atoms affect these parameters, and statistical methods are used to allow the control of the density parameters’ transferability.

We showed (Bak *et al.*, 2011 ▸) that there is no practical difference among the databanks in terms of their use as a source of aspherical scattering factors in TAAM refinement on X-ray data. All three databanks lead to a similar quality of structural data, and all have the same advantages over the IAM model. Quantitative comparison of the properties derived from the reconstructed densities, such as the deformation density, dipole moments, statistical characterizations of molecular electrostatic potentials, and interaction electrostatic energies (*E*_es_) for dimers of molecules, shows that the analysed databanks differ from each other. Comparison of *E*_es_ values with the results of quantum-chemistry calculations (at different levels) suggests that the UBDB is slightly better in this regard than other databanks. This may also mean that UBDB is better suited for electrostatic potential reconstruction.

Replacement of the IAM model by TAAM in the refinement procedure of X-ray diffraction data leads to more accurate geometrical information and provides access to quantitative estimation of the electron-density distribution and the properties derived from it (dipole moment, electrostatic potential *etc*.) for molecules in a crystalline environment. In comparison with IAM, TAAM refinement with X-ray data of atomic resolution (*d*_min_ = 0.7 Å) collected for crystals of small molecules (Bak *et al.*, 2011 ▸; Dominiak, 2014 ▸, and references cited therein):

(i) Improves the values of statistics describing the overall fit of the model to experimental data, for example, reducing the conventional *R* factors by about 1%, values of goodness of fit (GoF) becoming significantly closer to unity.

(ii) Reduces the values of the density maxima and minima, and randomizes their location on Fourier difference maps.

(iii) Significantly improves the position of the hydrogen atoms. For example, the resulting *X*—H bonds are longer by about 0.1 Å, and differ only by an average of 0.02 Å from the reference structures (from neutron diffraction data or those obtained from the optimization of the geometry of the periodic system).

(iv) Significantly improves the parameters of anisotropic atomic displacement parameters (ADPs) for non-hydrogen atoms. There is an approximately 10% reduction in the values of the ADPs relative to the IAM refinement.

Similar effects, though often on a smaller scale, were observed in the case of several X-ray data sets for macromolecular crystals (Dominiak, 2014 ▸; Malinska & Dauter, 2016 ▸, and references cited therein).

The UBDB was shown to give an excellent reproduction of the electron density and electrostatic potential for a number of amino acids, nucleobases and other organic molecules when compared with those calculated using conventional quantum-mechanics methods, while requiring only a small fraction of the computational time (Volkov, Li *et al.*, 2004 ▸; Volkov *et al.*, 2006 ▸; Kumar *et al.*, 2014 ▸; Volkov, Koritsanszky & Coppens, 2004 ▸; Czyżnikowska *et al.*, 2010 ▸; Bak *et al.*, 2011 ▸; Jarzembska & Dominiak, 2012 ▸). For molecules at equilibrium distance, the UBDB gave an error of *ca* 1.0 kcal mol^−1^ compared with quantum mechanics at the B3LYP/aug-cc-pVTZ level of theory. The UBDB method has been used to evaluate the electrostatic interaction energy for macromolecules such as the syntenin PDZ2 domain complexed with a set of four-residue peptides and the PDZ2 dimer (Dominiak *et al.*, 2007 ▸), the influenza neuraminidase interacting with a series of inhibitors (Dominiak *et al.*, 2009 ▸), various protein kinases interacting with the inhibitor sunitinib (Malinska *et al.*, 2014 ▸) and RNA with amino­glycosides (Kulik *et al.*, 2015 ▸).

It is also worth mentioning here some later-introduced alternatives to multipole refinement such as: X-ray wavefunction refinement (XWR) (Grabowsky *et al.*, 2012 ▸; Jayatilaka & Grimwood, 2001 ▸; Woińska *et al.*, 2017 ▸), molecular orbital occupation number (MOON) refinement (Hibbs *et al.*, 2005 ▸; Yang & Waller, 2013 ▸), libraries of extremely localized molecular orbitals (ELMO; Meyer *et al.*, 2016 ▸) and the maximum entropy method (MEM; van Smaalen *et al.*, 2003 ▸). Most recently, we have shown (Woińska *et al.*, 2016 ▸) that Hirshfeld atom refinement (HAR) (Jayatilaka & Dittrich, 2008 ▸), which constitutes one of the steps of XWR, is a very good alternative to TAAM refinement, especially for the systems for which quantum-mechanical calculations of mol­ecular wavefunctions are fast and easily accessible. There are also new developments in the context of pseudoatom modelling. To speed up intermolecular electrostatic interaction energy calculations we have recently introduced a new electron-density modelling technique called aug-PROmol (Bojarowski *et al.*, 2016 ▸). Another interesting model and database, called VIR, was proposed by Jelsch and co-workers (Nassour *et al.*, 2017 ▸).

Here we demonstrate that the interpretation of data from cryo-EM and ED methods can be based on the more realistic multipolar electron scattering model (TAAM) constructed with the help of UBDB. Better models lead to better experimental data fits and more accurate structural parameters such as *X*—H bond lengths or ADPs. We achieved this by performing crystal structure refinement with electron TAAM for a model compound, carbamazepine, for which several ED data sets have already been published, including the very recent one by Jones *et al.* (2018 ▸). In addition, high-quality sub-atomic resolution X-ray diffraction data and neutron diffraction data for carbamazepine are available which serve as external references to verify obtained results. To overcome the current experimental limits of ED data, we based our analysis mainly on theoretical ED data resulting from simulations done with the use of periodic density functional theory (DFT) calculations.

## 2. Methods   

*Theoretical background*. For high-energy (from a few keV to a few MeV) ED (scattering), the incident electrons can be effectively distinguished from the electrons of the solid (with energy in the order of less than 100 eV) (Peng, 1999 ▸). Then, the electrostatic potential, *V*(**r**), may be conveniently regarded as the scattering potential. *V*(**r**) may be expressed as
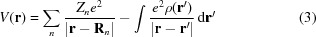
where the sum runs over atoms in the system, *e* is the elementary charge, *Z_n_* is the atomic number of the *n*th atom, **R**_*n*_ is its position and 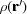 is the electron density at the point **r**′. The potential depends on both the positions of the nuclei and the distribution of the electrons around the nuclei via **R** *_n_* and 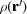 in equation (3), and it is this function which may in principle be retrieved from elastic electron scattering experiments. Further, we may conveniently express the electron density in terms of the sum of individual atoms centred at the positions **R** *_n_*. Thus,
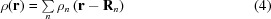
and therefore
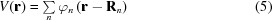
with
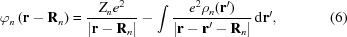

*i.e*. the total scattering potential may be expressed as a superposition of contributions from individual atoms.

The atomic scattering factor for the elastic scattering of electrons [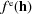] is proportional to the Fourier transform of the electrostatic Coulomb potential of an atom 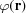, *i.e*.
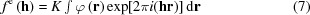
where *K* is a multiplier and (in SI units) *K* = 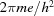 = 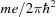, *m* and *e* are the mass and charge of the electron, respectively, 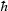 = *h*/2π where *h* is the usual Planck constant, and |**h**| = 2 sin θ/λ, in which θ is one half of the scattering angle and λ is the electron wavelength. Relativistic values of *m* and λ are assumed (Cowley *et al.*, 2006 ▸).

By the use of the Poisson equation relating the potential and charge-density distributions
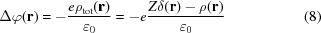
where 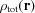 is total charge (nuclei and electron) density and 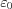 is the permittivity of a vacuum, it is possible to derive the Mott–Bethe formula for 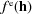 in terms of the atomic scattering factors for X-rays, 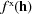 (Bethe, 1930 ▸; Mott, 1930 ▸; Peng, 1999 ▸):
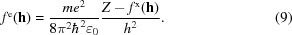



Equation (9) conveniently may be used to convert known X-ray atomic scattering factors from any model of atomic electron densities to electron scattering factors.

*Reference structure*. A crystal structure of form III of carbamazepine from neutron diffraction studies performed at 100 K (Sovago *et al.*, 2016 ▸) served as the major reference in our studies.

*Structure factors*. In general we used four different sets of structure factors: experimental X-ray diffraction structure-factor (xSFex) amplitudes, 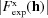, with resolution up to *d*_min_ = 0.42 Å (Sovago *et al.*, 2016 ▸), experimental ED structure factors (eSFex) amplitudes, 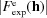, up to *d*_min_ = 0.6 Å (Jones *et al.*, 2018 ▸), theoretical X-ray structure factors (xSFth), 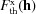, computed up to *d*_min_ = 0.38 Å from crystal periodic wavefunctions and including atomic motions at 100 K determined by neutron diffraction (Sovago *et al.*, 2016 ▸), and theoretical electron structure factors (eSFth), 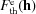, computed from theoretical X-ray structure factors by application of the Mott–Bethe formula.

To obtain theoretical structure factors, the experimental geometry of the carbamazepine crystal (van Genderen *et al.*, 2016 ▸) was optimized by applying periodic DFT calculations implemented in *CRYSTAL14* (Dovesi *et al.*, 2014 ▸, 2016 ▸). Optimization was performed by the B3LYP-D* method, in which B3LYP was augmented with an empirical dispersion term as proposed by Grimme (2006 ▸) and modified for mol­ecular crystals (Civalleri *et al.*, 2008 ▸). The DZP basis set (Dunning, 1970 ▸) was used. A full, simultaneous relaxation of both lattice parameters and atomic coordinates by means of analytical energy gradients was applied. The level of accuracy in evaluating the Coulomb and exchange series was controlled by five TOLINTEG parameters for which values of 10^6^, 10^6^, 10^6^, 10^7^, 10^25^ were used. The DFT exchange-correlation contribution was evaluated by numerical integration over the cell volume. Radial and angular points of the atomic grid were generated through Gauss−Legendre and Lebedev quadrature schemes. The condition for the self-consistent field (SCF) convergence was set to 10^8^ on the total energy difference between two subsequent cycles. The shrinking factors (IS) along the reciprocal-lattice vectors were set at 4. The level shifter value was set to 0.6 Hartree. A Fock (Kohn–Sham) matrix mixing of 30% between subsequent SCF cycles was used. The total energies obtained with this mesh were fully converged. The crystal symmetry was imposed as a constraint during the whole optimization process. Upon energy convergence the periodic wavefunction was obtained.

The optimized unit cell was 3.2% smaller than the one obtained from neutron diffraction experiments at 100 K (Table S1 in the supporting information) and 2.6% and 2.3% smaller than from X-ray and ED, respectively. The *X*—H bond lengths differed slightly between the optimized structure and that from neutron diffraction. These were not one direction differences as optimized bond lengths were between 0.057 Å shorter and 0.041 Å longer. The root-mean-square difference (RMSD) between the lengths obtained from neutron diffraction and periodic optimization was 0.033 Å and the mean error (ME) was 0.011 Å.

Optimized structure static and dynamic theoretical structure factors for X-ray diffraction were computed, up to sin θ/λ = 1.3 Å^−1^, in *CRYSTAL14*. Please note that in this work the term ‘static structure factors’ indicates structure factors computed for frozen atoms, and ‘dynamic structure factors’ means structure factors which contain contributions from atomic motions due to thermal vibrations. In the case of dynamic structure factors we made an attempt to compute dynamic structure factors with theoretical anisotropic ADPs computed in *CRYSTAL14* (Dovesi *et al.*, 2014 ▸, 2016 ▸) from vibrational frequencies. The temperature was set to 100 K. ADPs were obtained based on harmonic frequencies at the Γ-point (Pascale *et al.*, 2004 ▸) and were computed with the same settings as used for structure optimization. Some frequencies are equal to zero at the Γ-point and vary significantly away from it; such ADPs are usually smaller when compared with ADPs from refinement against neutron diffraction data. Here it appeared they were too small for our purposes (Fig. S1), being 1.65 times the size of the reference for non-H atoms and 1.34 times the size of the reference for H atoms. The shapes of atomic displacement ellipsoids in both sets were roughly similar. Finally we used ADPs taken from a neutron diffraction structure (Sovago *et al.*, 2016 ▸) to compute dynamic X-ray structure factors in *CRYSTAL14* (Erba *et al.*, 2013 ▸).

Electron structure factors, 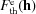, were computed from X-ray structure factors, 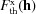, by applying the Mott–Bethe formula to the structure-factor expression which gives the following relation:
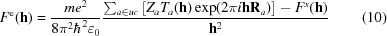
where the summation runs over all atoms in the unit cell (*uc*) and 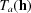 are the temperature factors. The functionality enabling this calculation was implemented by us using the DiSCaMB library (Chodkiewicz *et al.*, 2018 ▸).

All theoretical structure factors were reported on an absolute scale. 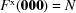 where *N* is the number of electrons in the unit cell and was equal to 496 e for the studied crystal, while 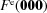 was set to equal to 201.573 Å and includes information about the mean electrostatic potential in the unit cell divided by unit-cell volume. Thus the mean electrostatic potential of the carbamazepine crystals was estimated to be 8.7 V [*F* ^e^(**000**)(Å) /Volume (Å^3^) multiplied by 47.8780 to bring the potential to volt units, see ch. 4.3.1.7 in *International Tables for Crystallography* Vol. C (Wilson & Geist, 1993 ▸)]. All 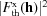 were associated with 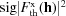 = 1.0 (sig is an estimated standard deviation) and all 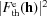 with 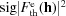 = 0.01 so that the number of reflections fulfilling the criterion 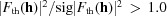 were comparable (*ca* 98%).

*Structure solution*. All structures were solved independently in *Olex2* (Dolomanov *et al.*, 2009 ▸), with the use of different methods: intrinsic phases implemented in *SHELXT* (Sheldrick, 2015 ▸) in the case of the experimental X-ray data set and charge flipping implemented in *olex2.solve* for the remaining cases. We transformed (*WinGX*; Farrugia, 2012 ▸) atomic coordinates and structure factors to standardize the unit cell and the asymmetric unit in all cases.

*Structure refinement*. A locally modified version of *Olex2* version 1.2 (Dolomanov *et al.*, 2009 ▸) was used for the refinements. It incorporates the IAM and TAAM models of X-ray (xIAM and xTAAM) or electron scattering factors (eIAM and eTAAM, respectively) available in the DiSCaMB library (Chodkiewicz *et al.*, 2018 ▸) into the *olex2.refine* module. For X-ray IAM parameterization from Tables 4.2.6.8 and 6.1.1.4 of *International Tables for Crystallography* Vol. C (Wilson & Geist, 1993 ▸) was used. For electron IAM parameterization from Table 4.3.2.3 of *International Tables for Crystallography* Vol. C (Cowley *et al.*, 2006 ▸) was used. To parameterize TAAM, the newest version (Kumar *et al.*, 2019 ▸) of the UBDB databank (Volkov, Li *et al.*, 2004 ▸; Dominiak *et al.*, 2007 ▸; Jarzembska & Dominiak, 2012 ▸) was used together with *LSDB* (Volkov, Li *et al.*, 2004 ▸). The parameters were transferred after the first cycles of the IAM refinements done during the structure solution step. The Mott–Bethe formula was used to obtain electron form factors from the Hansen–Coppens multipolar model (Hansen & Coppens, 1978 ▸) representation based on those originally calculated for X-ray data as described previously (Chodkiewicz *et al.*, 2018 ▸).

All refinements were performed against |*F*(**h**)|^2^ using the Gauss–Newton minimization method. Chemical occupancies were fixed to 1.0. No correction for extinction was used. For most of the refinements the following weighting scheme was used:
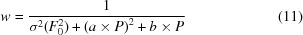
where *P* = 1/3 × maximum of (0 or 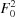) + (1 − 1/3) × 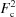. Optimal values for the *a* and *b* parameters were automatically calculated in order to achieve normal distribution of residuals. We also tested a weighting scheme with *a* and *b* parameters equal to zero (which gives a statistical weighting scheme for experimental data and effectively a unit weighting scheme for simulated data); however, such refinements, especially against ED structure factors (eSF), were less stable and gave worse fitting parameters and geometry [*e.g.* many non-positive definite (NPD) ADPs]. The results of such refinements are presented in Table S3.

IAM and TAAM refinements were performed gradually. For dynamic X-ray data sets (xSFex and xSFth) only the atomic coordinates and isotropic ADPs (*U*_iso_) for non-H atoms, and H atoms constrained by the appropriate AFIX commands [AFIX 93 for H(NH2-planar) and 43 for H(C—H-aromatic)] with the *X*—H bond lengths of 0.86 Å and 0.93 Å, respectively, were used for refinement (option 1), then anisotropic ADPs (*U*_aniso_) for non-H atoms were included (option 2), atomic coordinates and *U*_iso_ for H atoms (not restrained) (option 3) and finally anisotropic ADPs for H atoms (option 4). For dynamic electron data sets (eSFex and eSFth) the steps of refinements were the same with the exception of constraining the bond lengths to H-atom parameters. The bonds were restrained to the average neutron diffraction values (Allen & Bruno, 2010 ▸) with an e.s.d. = 0.02 Å [DFIX commands instead of AFIX: DFIX 1.01 Å for H(NH2-planar) and DFIX 1.083 Å for H(C—H-aromatic)]. The values of *U*_iso_ parameters for H atoms in options 1 and 2 were constrained to the values computed from *U*_iso_/*U*_aniso_ of their covalent partner, in exactly the same way as was done in the case of X-ray data. Refinements resulting in NPD ADPs for most of the atoms (for eSFex 0.8 Å options 2–4 and 0.6 Å option 4) were excluded from our comparison. For X-ray data sets gradual refinements were not indispensable, because refinements were quite stable. But for electron data sets this helped to find the best structure, because refinements were generally less stable and more steps were needed to locate the minimum. A short summary of refinement options is given in Table 1 ▸ for further reference.

We also performed test refinements against ED data with *X*—H bonds constrained to distances expected for X-ray radiation (regular AFIX command). The results clearly showed that such constraints were very inadequate for ED (see Table S2).

Refinements with various resolution cut-offs were performed: *d*_min_ = 0.83 Å [minimum resolution for small molecules accepted by the International Union of Crystallography (Spek, 2003 ▸)], *d*_min_ = 0.60 Å (resolution where the valence electrons of light elements have a negligible contribution to X-ray scattering factors), and the maximum available resolution (*d*_min_ = 0.38 Å for theoretical data sets, and *d*_min_ = 0.42 Å for the experimental X-ray data set).

*Electrostatic potential and electron density of a single molecule*. Electrostatic potentials and electron densities of a single carbamazepine molecule were computed from TAAM and IAM* using the *XDPROP* module of the *WinXD2016* package (Koritsanszky *et al.*, 2003 ▸; Volkov *et al.*, 2016 ▸). The IAM* was built from the Hansen–Coppens model with all parameters set to the values corresponding to neutral spherical atoms including H atoms. Cubic grids with a 0.05 Å step size and 300 × 300 × 300 grid points were used. The reference electrostatic potential and electron-density grids were calculated for a single carbamazepine molecule in a vacuum using *GAUSSIAN09* (Frisch *et al.*, 2009 ▸) at the B3LYP/cc-pVDZ level of theory (Krishnan *et al.*, 1980 ▸; Becke, 1988 ▸; Perdew, 1986 ▸; Lee *et al.*, 1988 ▸). Grids were plotted in *MoleCoolQt* (Hübschle & Dittrich, 2011 ▸).

*Fourier maps*. Fourier maps were calculated and plotted in *MoleCoolQt* (Hübschle & Dittrich, 2011 ▸). Input xd.fou files [format specific for the *WinXD2016* package (Koritsanszky *et al.*, 2003 ▸; Volkov *et al.*, 2016 ▸)] were prepared with the help of locally developed software. The values on density maps computed from electron structure factors were multiplied by 3.32494 to bring them to e Å^−1^ units of electrostatic potential. The multiplier results from application of the procedure recommended in ch. 4.3.1.7. of *International Tables for Crystallography* Vol. C (Wilson & Geist, 1993 ▸) allowed us to achieve volt units of electrostatic potential followed by conversion of volt to e Å^−1^ units.

## 3. Results   

### 3.1. Models of electrostatic potential   

In order to discuss any results regarding the performance of various models in a proper description of electron scattering, it is very instructive to firstly examine how the electrostatic potential and electron density of a single carbamazepine molecule look according to direct DFT calculations, or TAAM and IAM simulations. This will give us a proper starting point for further analysis.

**Figure 1:**
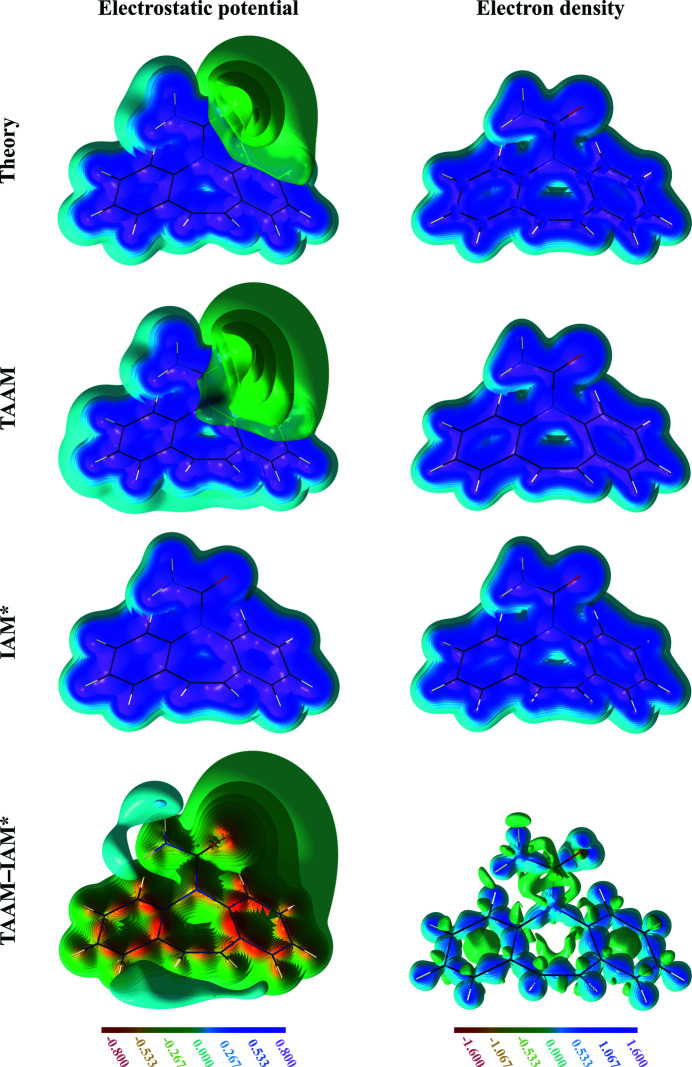
Electrostatic potential (e Å^−1^; left) and electron density (e Å^−3^; right) for an isolated carbamazepine molecule computed from various models: the theoretical wavefunction at the B3LYP/cc-pVDZ level, TAAM, IAM* and the TAAM − IAM* difference. The properties were computed directly from the models without passing through Fourier transformations. Contours are every 0.05 e Å^−1^ up to ±0.80 e Å^−1^ for the electrostatic potential, and every 0.1 e Å^−3^ up to ±1.6 e Å^−3^ for the electron density.

The electrostatic potential of the carbamazepine molecule (Fig. 1 ▸) has clear regions of negative potential surrounding the oxygen atom, where the lone electron pairs are expected to be localized. The potential reaches values of −0.21 e Å^−1^ according to the B3LYP/cc-pVDZ method. The negative feature and the shape of the entire positive potential, as defined by the low-value iso-contours, seem to be very well reproduced by TAAM (with *V*_min_ = −0.22 e Å^−1^). When the potential from IAM is analysed, it is clearly visible that it lacks any information on negative potential regions. Closer inspection of the potential difference, done with the help of the TAAM − IAM deformation potential map, reveals that there is a systematic bias in the potential description by IAM, spanning over the whole molecule, not only accumulating where the negative potential is expected. The errors are larger, on an absolute scale, than the minimum value of the potential in the negative potential region. Values of the deformation potential range from *V*_min_ = −0.793 e Å^−1^ to *V*_max_ = 0.104 e Å^−1^. Generally, the IAM potential is too positive in both the electron pair regions and in the covalent bonding regions, and not positive enough in the regions surrounding the polar H atoms. All the observations are similar to those we already noted among many other organic molecules (Kumar *et al.*, 2019 ▸).

When the electrostatic potential maps were compared with electron-density maps, it could be observed that the apparent shape of a molecule, as defined by its low-value iso-contours, was different. In the case of the exact electrostatic potential, H atoms are noticeably separated from their covalent neighbours, whereas the electron densities of the H atoms were more fused with their covalent neighbours. In general, all the atoms and bonds appeared to be better separated from each other on the exact electrostatic potential map than on the exact electron-density map. The IAM models give a false description of the molecular density shapes, and the discrepancy seems to be more pronounced in the case of the electron density. The appearance of the TAAM − IAM electron-density deformation map is different to that for the electrostatic potential. The deformation electron density is less diffuse and mainly localized in-between atoms or at positions of electron pairs. The values of the electron deformation density range from ρ_min_ = −0.941 e Å^−3^ to ρ_max_ = 0.984 e Å^−3^.

All of the above will have consequences for structure refinement against electrostatic potential as compared with the electron density, and will have different effects when IAM is replaced by TAAM in these refinements.

### 3.2. Structure factors from various models   

The discrepancies observed in real space for the electrostatic potential and electron density computed from various models should also be visible in reciprocal space. Indeed, analysis of the structure-factor amplitudes computed from the IAM and TAAM models applied to the carbamazepine crystal structure reveals that there are regions in reciprocal space in which the IAM significantly varies from the reference values obtained from periodic quantum-mechanical calculations. The highest discrepancies between the IAM model and the reference are observed in the low-resolution range.

**Figure 2:**
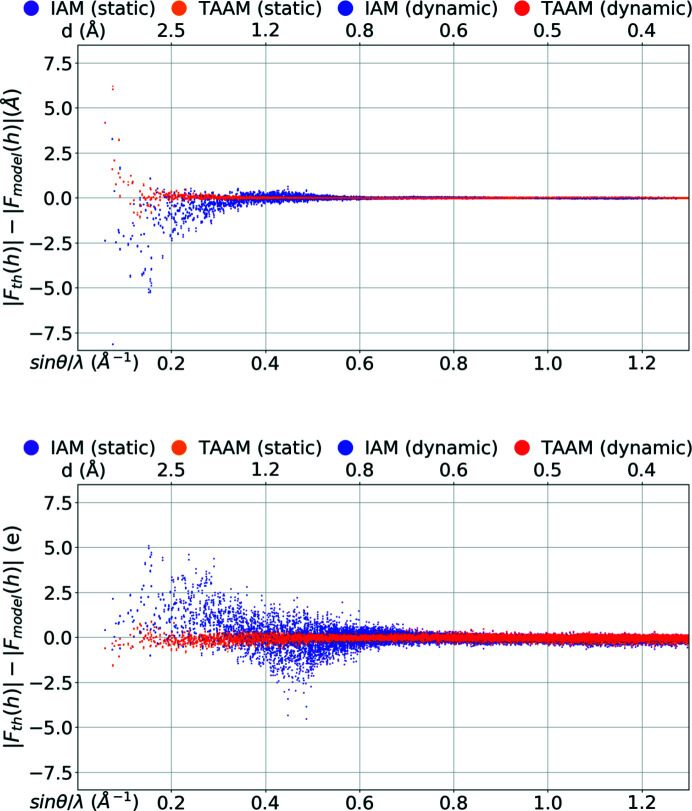
|*F*_th_(**h**)| − |*F*_model_(**h**)| versus resolution [sin θ/λ (Å^−1^), *d* (Å)] for the IAM and TAAM models applied to the target crystal structure: (top) electron |*F*(**h**)| (Å), (bottom) X-ray |*F*(**h**)| (e). |*F*(**h**)| were computed using the target atomic positions and thermal parameters (dynamic) or with target atomic positions only (static).

In the case of ED, the amplitudes of structure factors computed with IAM are mostly too large in the region from *d* = 8.5 Å up to *d* ≅ 1.5 Å (Figs. 2 ▸ and 3 ▸, and Figs. S3 and S4 in the supporting information). *d* = 8.5 Å represents the lowest-resolution (lowest spatial frequency *d* ^−1^) reflections observable for crystal structures of carbamazepine form III. Differences increase quickly with decreasing resolution. On an absolute scale, the differences in the amplitudes of individual reflections reach values up to 8.1 Å for dynamic structure factors. On a relative scale, the 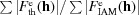 ratio computed from the entire group of dynamic structure factors in the 8.5–1.5 Å resolution range was 0.92 and the 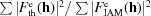 ratio was 0.81. Finally, the value of the *R*1 factor for IAM was as high as 10%. In the resolution range from *d* ≅ 1.5 Å to *d* ≅ 0.9 Å, the IAM amplitudes are, on the other hand, mostly too small, but the differences were not large. The 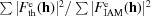 ratio was 1.02 for *d* ≅ 1.0 Å and the *R*1 was 2.3%. From *d* ≅ 0.9 Å up to *d* = 0.38 Å, the differences were almost invisible on an absolute scale. However, on a relative scale they were still noticeable and oscillated over the range of resolutions. The values of the 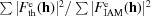 ratio were between 1.01 and 0.98, and of the *R*1 factors between 0.7% and 1.5%.

The amplitudes resulting from IAM using the X-ray diffraction results were too small in the lowest-resolution range, *i.e.* up to *d* ≅ 1.5 Å. Similarly to the electron scattering factors, the differences rose at worse resolutions and reached values of 5 e for the dynamic data set. However, for the X-ray data, the differences were smaller on a relative scale: the 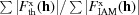 ratio was 1.05 and the 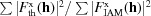 ratio was 1.10, and the *R*1 factor was 5.6% for the dynamic structure factors. In the resolution range from *d* ≅ 1.5 Å to *d* ≅ 0.6 Å, the IAM amplitudes were either too small or too large, the latter slightly dominating at *d* ≅ 1.0 Å and the former more often at *d* ≅ 0.8 Å. On an absolute scale the differences were smaller than at lower resolutions, but the drop in difference values was not as large as it was for ED. The 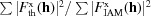 ratio was 0.95 for *d* ≅ 1.0 Å and 1.01 for *d* ≅ 0.8 Å. The *R*1 was still high at *d* ≅ 1.0 Å, 5.4%, and at *d* ≅ 0.8 Å was 2.8%. At higher resolution the ratio became smaller and smaller. At *d* ≅ 0.67 Å the 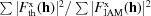 ratio was 1.00 and the *R*1 was 1.4% for the dynamic structure factors. Then the ratio decreased further, up to 0.97, and the *R*1 value began to rise. *R*1 rose faster for dynamic structure factors than for static ones and at *d* ≅ 0.38 Å it reached a value of ∼3% for dynamic and 1.7% for static structure factors. The last observation was the only significant discrepancy in the behaviour of the IAM amplitudes in relation to the exact values, depending on whether the thermal motion model was included in the structure-factor calculations (dynamic) or not (static). All other observations were the same for the static and for the dynamic structure factors.

To sum up, in the low-resolution region (up to *d* ≅ 1.5 Å) the IAM amplitudes for ED were too large while they were too small for X-ray. The departure of the IAM amplitudes from the exact values for ED data was larger on a relative scale when compared with X-rays, but it disappeared faster with rising resolution.

The usage of the TAAM model in structure-factor calculations significantly lowered the discrepancies of the model amplitudes from the exact theoretical reference values in the entire resolution range (*d* = 8.5–0.38 Å) in the case of ED, and in resolution up to *d*_min_ ≅ 0.67 Å for X-rays, when compared with IAM.

In the case of ED, the TAAM model gave excellent agreement with the exact structure-factor amplitudes almost for the entire resolution range studied. The 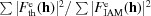 ratios were in the range of 1.000 to 1.005, and the *R*1 factors were between 0.5% and 0.7% for dynamic structure factors, except for resolutions lower than *d* ≅ 3 Å. At *d* ∼< 3 Å the TAAM amplitudes significantly departed from the exact ones, but the discrepancy was not as high as for IAM. For the resolution bin of *d* = 8.6–1.4 Å, the 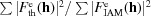 ratio was 1.03, and the *R*1 factor was 2.3%.

In the case of X-ray diffraction, TAAM agreed very well with exact amplitudes at the resolution range of *d* = 8.6 Å to *d* ≅ 0.67 Å, which included the lowest-resolution bin as well. The 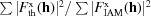 ratios were in the range of 0.991 to 1.000, and the *R*1 factors were between 0.9% and 1.1% for dynamic structure factors. At the highest-resolution range TAAM behaved similarly to IAM. Amplitudes from both models started to deviate from the reference, and the discrepancies were even more enhanced when thermal motions were included in the amplitude calculations. It is interesting to observe that this effect was not visible for ED at the same resolution range.

The above analysis confirmed that the differences in the electrostatic potential computed from the TAAM and IAM models in real space were also significant in reciprocal space in resolution ranges that can now be collected experimentally from small-molecule crystals. During refinement, the expected drop in the overall *R*1 factor when IAM was replaced by TAAM was of a similar magnitude in the case of the ED as was observed in the X-ray data set (Table 2 ▸). We may also speculate that in the case of refinements of macromolecular crystal structures against data sets of lower than *d*_min_ = 0.83 Å resolution, the drop will be higher for ED data than for X-ray. This however must be investigated further.

### 3.3. Fourier density maps from various models   

Diffraction experiments are always limited by resolution (minimal and maximal). In addition, atoms undergo thermal motion, which also influences the observed density. It is interesting to compare potential density maps (and electron-density maps, including thermal parameters typical for 100 K) for studied molecules, computed with various models, with those from Fourier summation with typical resolution ranges.

**Figure 4:**
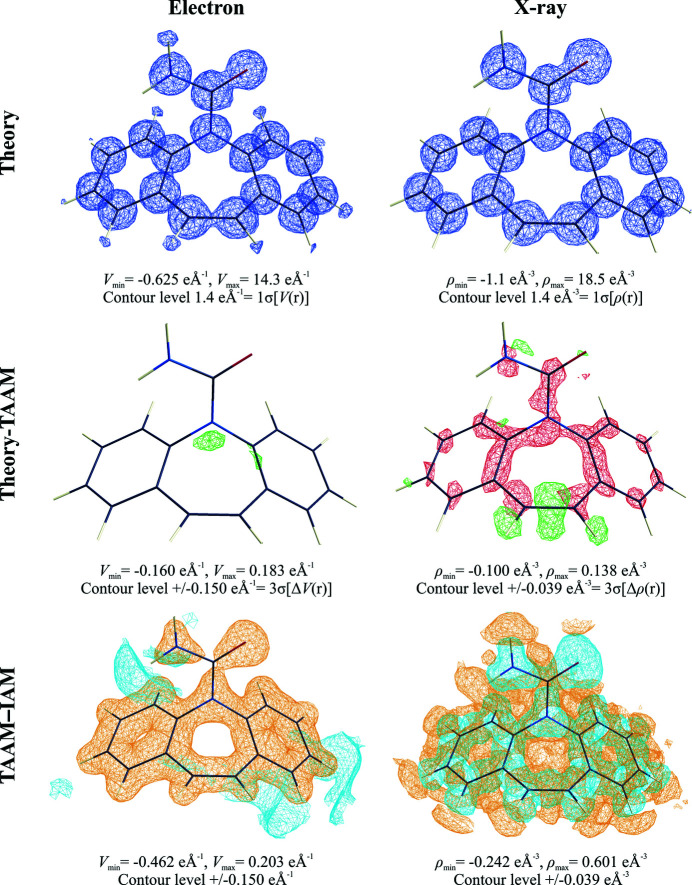
Fourier density maps computed from electron (e Å^−1^) and X-ray (e Å^−3^) dynamic structure factors of the carbamazepine crystal, truncated at *d*_min_ = 0.83 Å. Structure factors were computed for target atomic positions and thermal parameters. Theory: the 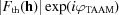 map; Theory–TAAM: the 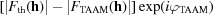 map using green contours for positive density and red for negative; TAAM–IAM: the 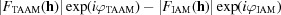 map using blue contours for positive density and orange for negative.

The 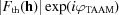 electrostatic potential map (Fig. 4 ▸), which simulates the *F*_obs_ map commonly analysed during structure solution and refinement steps for experimental ED data analysis, clearly shows that H atoms can in fact be visible at standard resolution, *d*_min_ = 0.83 Å, and at 100 K. This is not the case for X-ray diffraction electron-density maps, as confirmed by our simulation.

When TAAM is applied, there is little left on the 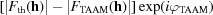 residual potential map. Indeed TAAM very well models reference diffraction data. The 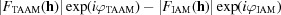 deformation potential map, contoured at the same level as the residual map {3σ[Δ*V*(**r**)]}, clearly confirms that the limitations of IAM are obvious and cause the appearance of a strong negative signal {which is up to 9σ[Δ*V*(**r**)]}. The signal was less strong, and of an opposite sign when compared with X-ray diffraction {which is up to ∼15σ[Δρ(**r**)]}. With increasing resolution, all the above observations still hold true; however the features on the maps become smoother and a little more detailed.

The residual electron-density maps from X-ray amplitudes also illustrate where differences in structure-factor amplitudes (see below) localize in real space. They were placed in close vicinity to nuclear positions. On residual maps computed from dynamic structure factors the residual electron density is concentrated mostly in negative blobs which at higher resolution appeared to be a part of quadrupole-like residual densities. On residual maps (Fig. S7) computed from static structure factors they emerged as sharp spherical minima. This is a well-known phenomenon, observed in many quantum-crystallography studies [see Kumar *et al.* (2018 ▸) and references cited therein]. The minima are most often explained by electron core contractions occurring due to covalent bond formation, which is not taken into account in IAM nor in TAAM. Apparently, discrepancies in the modelling of thermal motions by the Mulliken partition in *CRYSTAL14* and by pseudoatom partition in the TAAM refinement result in the appearance of aspherical deformations of the originally spherical residual peaks. It is very interesting that the residuals concentrated close to nuclei are not visible in Fourier potential maps computed from electron scattering factors.

**Figure 5:**
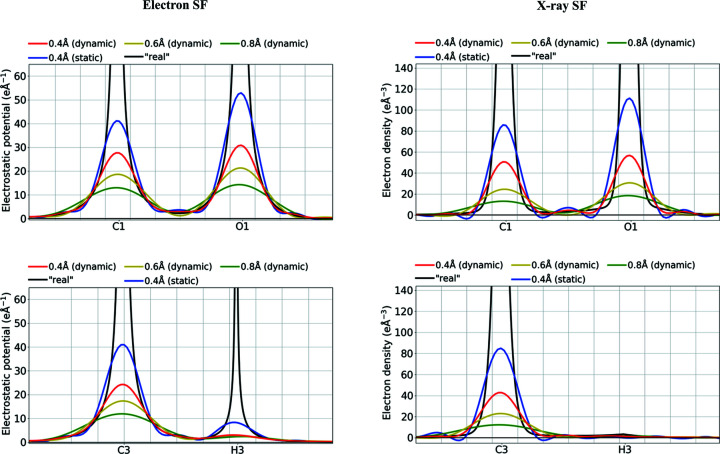
Fourier density maps [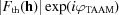] of the electrostatic potential (e Å^−1^) or electron density (e Å^−3^) along selected covalent bonds computed from electron and X-ray dynamic or static structure factors of the carbamazepine crystal, truncated at various resolutions (Å). ‘Real’ values computed directly from TAAM models without passing through the Fourier transformations are given for reference. Values were computed for target atomic positions and thermal parameters.

It also interesting to compare the shapes of individual atomic density peaks. The real electrostatic potential peaks are much wider at the bottom of the peak than the electron-density peaks (Fig. 5 ▸), and much narrower closer to the nuclei (Fig. S8). Nevertheless, the widths of the same peaks on Fourier maps computed for the studied resolution ranges seem to be more similar. The non-H-atom density peaks for potentials are only *ca* 5% wider than the electron-density peaks at half peak heights at low resolution. The difference increased with increasing resolution and was up to *ca* 30% for the highest resolution examined here.

### 3.4. Crystal structure refinements with various models   

The main goal is to check whether eTAAM fits ED data more accurately than eIAM, and improves structural information (geometry and thermal motion description) obtained through structure refinement against experimental ED data. Neutron and X-ray diffraction data, as well as results presented in Sections 3.2 and 3.3, will provide reference points for this analysis.

Because the quality of the experimental ED data set (eSFex) is not yet good enough for such detailed analysis, we will draw conclusions mainly on the basis of refinements against the theoretical data set (eSFth). However we will also show results based on experimental data, whenever possible, to present the current limits of the method.

We will check if our conclusions are independent of data resolution or, the opposite, if they are valid only for a particular resolution range. We will also check how the constraints and/or restraints imposed on thermal motion parameters and on H-atom positions influence the results.

#### 3.4.1. Validity of usage of simulated theoretical structure factors for crystal structure refinements   

To simulate crystal structure refinement against diffraction data, which are of high resolution and uncontaminated by errors, we can use theoretical structure factors computed from periodic wavefunctions. The question then becomes one of verifying the ‘correctness’ of the simulation. We can evaluate this by the comparison of theoretical and experimental high-resolution data from X-ray diffraction, because high-quality experimental data are available and because there is a vast accumulated knowledge in the literature regarding the behaviour of refinements of various electron-density models with sub-atomic resolution X-ray data.

**Figure 6:**
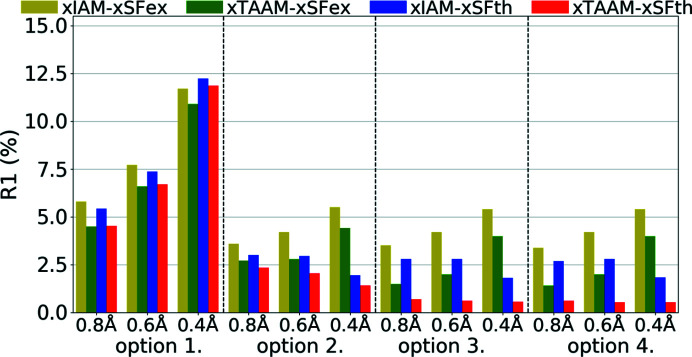
Overall *R*1 agreement factors (%) for structure refinements with experimental (xSFex) or theoretical (xSFth) X-ray diffraction data sets truncated to various resolutions (*d*_min_ = 0.8, 0.6, 0.4 Å) using various scattering models (xIAM, xTAAM) and sets of refined parameters (options 1, 2, 3 or 4).

It appeared that both xIAM and xTAAM structure refinements with theoretical X-ray diffraction data (xSFth) achieved *R*1 values of similar magnitudes as compared with experimental data (xSFex) (Fig. 6 ▸, Table S3). The values were in the range of 1–12% and they obviously decreased when a less constrained set of refined structural parameters was used. On the other hand, the *R*1 values increased with increasing resolution. It might be noted, however, that the increase with resolution was less for theoretical data, except for the most constrained parameter set (option 1).

The xTAAM refinements gave lower *R*1 values than xIAM for both xSFth and xSFex for all refinement options and resolutions. For both xSFth and xSFex, the improvement was more visible when fewer constraints were used. For both xSFth and xSFex, the largest improvement was observed for medium resolution. It is interesting to note that, despite the common understanding of how much information about H atoms can be retrieved from X-ray diffraction data, refinements with option 4 were stable and led to acceptable values of the refined parameters (as will be shown below) for almost all types of refinement.

It is interesting to note that all *R*1 values achieved by refinement with xSFth and option 4 were smaller than the ones computed for target atomic positions and anisotropic ADPs (Table 2 ▸). On an absolute scale the differences were not negligible. The largest one, 2.15%, was for xIAM at low resolution. Apparently, refinement of the model parameters (atom positions, ADPs) and optimization of the *a* and *b* parameters of the weighting scheme lead to incorrect values, which artificially compensated for the limitations of static density and thermal motion models.

**Figure 7:**
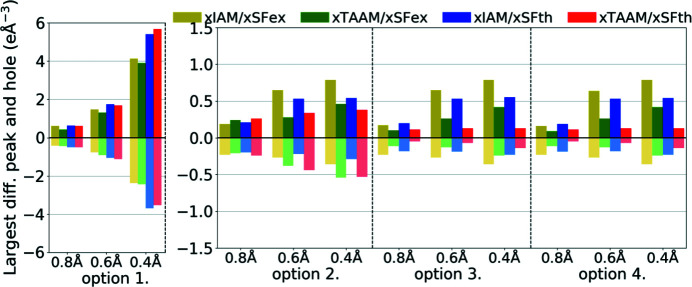
Largest difference peak and hole (e Å^−3^) on the residual density Fourier maps for structure refinements with experimental (xSFex) or theoretical (xSFth) X-ray diffraction data sets truncated to various resolutions (*d*_min_ = 0.8, 0.6, 0.4 Å) using various scattering models (xIAM, xTAAM) and sets of refined parameters (options 1, 2, 3 or 4).

Maximum residual densities (Fig. 7 ▸, Table S3), another measure of the quality of fit, depend on the resolution and the refinement option. Trends in xTAAM improvements over xIAM were the same for theoretical and experimental data sets when options 2, 3 and 4 were applied.

Residuals obtained after refinement were always lower than residuals computed for models with target values (Fig. 4 ▸ and Figs. S5–S6). The differences were larger for xIAM than for xTAAM. This may have been due to compensating for model limitations by refinement of model parameters (coordinates and ADPs) to incorrect values supported by the optimized weighting scheme.

The weighting parameters *a* and *b* behave similarly during refinements against xSFth and xSFex. Both for xSFth and xSFex we observed that refinements with option 1 have enormous *b* parameters, which might be a way of hiding problems with too simple a model. After excluding option 1 we see higher values of *a* and *b* for the xIAM model than for the xTAAM model.

**Figure 8:**
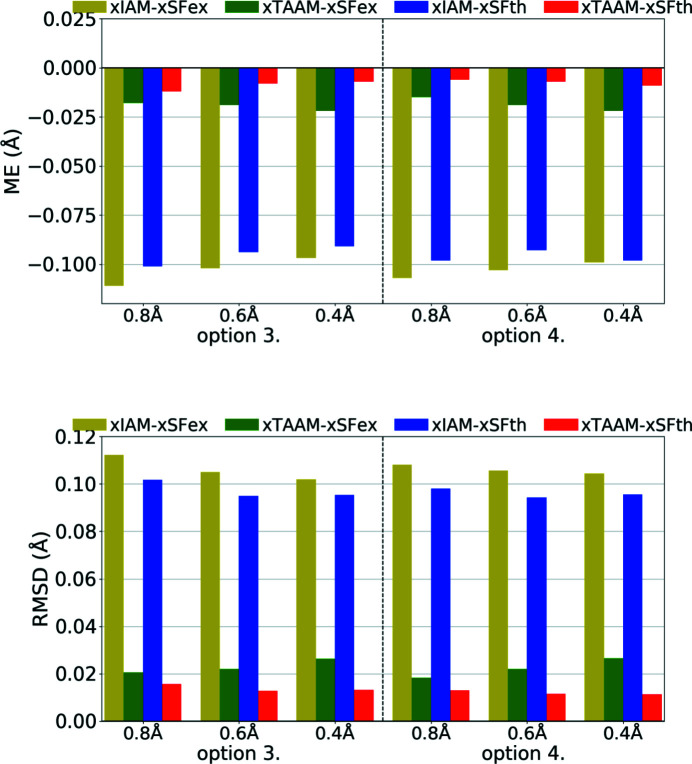
The mean error (ME) and the root-mean-square difference (RMSD) of the *X*—H bond lengths (Å) for structure refinements with experimental (xSFex) or theoretical (xSFth) X-ray diffraction data sets truncated to various resolutions (*d*_min_ = 0.8, 0.6, 0.4 Å) using various scattering models (xIAM, xTAAM) and sets of refined parameters (options 3 or 4). The statistics were computed with reference to values from neutron diffraction collected from the carbamazepine crystal. The ME is defined as 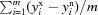, and the RMSD is defined as 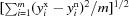 where 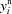 is the reference value from neutron diffraction and 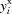 the value from refinement with X-ray data.

The *X*—H bond lengths, obtained during refinements with options 3 and 4, were similar for the same scattering models (Fig. 8 ▸, Table S3). All bond lengths were shorter than from neutron diffraction. For xIAM the shortening was substantial and independent of resolution, and the ME was around −0.10 Å for both theoretical and experimental data. When using xTAAM, the lengths increased. The ME for xTAAM was around −0.01 Å for theoretical data sets and around −0.02 Å for experimental data. The difference between xIAM and xTAAM could be easily noticed with the use of theoretical X-ray diffraction data and the level of improvements was the same as for experimental data.

**Figure 9:**
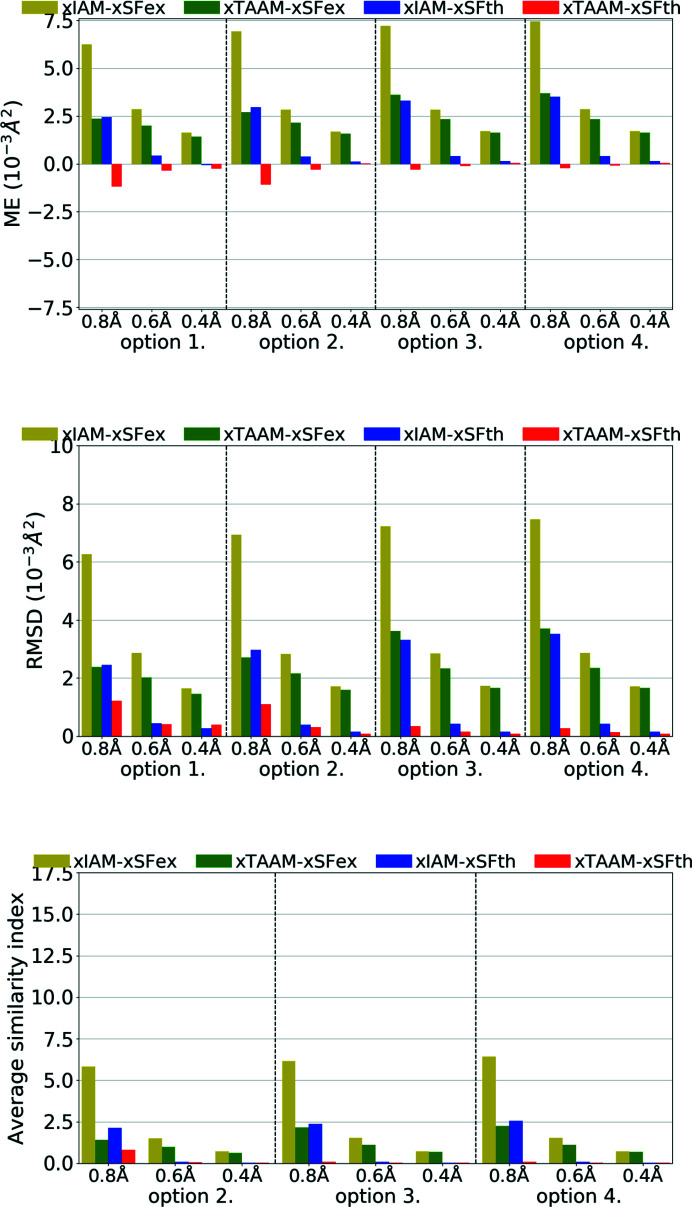
The mean error (ME) and the root-mean-square difference (RMSD) of *U*_iso_/*U*_eq_ (10^−3^ Å^2^), and the average similarity index of anisotropic ADPs for non-H atoms for structure refinements with experimental (xSFex) or theoretical (xSFth) X-ray diffraction data sets truncated to various resolutions (*d*_min_ = 0.8, 0.6, 0.4 Å) using various scattering models (xIAM, xTAAM) and sets of refined parameters (options 1, 2, 3 or 4). The indicators were computed with reference to values from neutron diffraction collected from the carbamazepine crystal.

Another parameter to be compared is the ADPs. Refinement against theoretical X-ray diffraction data gave non-H-atom thermal ellipsoids that were more similar in size and shape to the reference ones from neutron diffraction than from refinements against experimental data (Fig. 9 ▸, Table S3). This is understandable as neutron values are exact target values for theoretical data. Nevertheless, trends were the same for the theoretical data set as for the experimental one. The errors of the ADPs became smaller with improved resolution for both xSFth and xSFex although more significant changes were observed for xIAM than for xTAAM. The usage of xTAAM improved the accuracy of non-H *U*_iso_/*U*_eq_ when compared with xIAM. The improvement was very high, around 25–35% of the reference value for the low-resolution data set. With improving resolution the positive effect of using a more accurate scattering model was less pronounced, but still bigger than the mean e.s.d.s of *U*_iso_/*U*_eq_ (Table S3).

To analyse the shape of thermal ellipsoids we used similarity index *S*_12_ (Whitten & Spackman, 2006 ▸) which measures the spatial overlap of two probability density functions defined by two sets of ADPs. It appeared that for theoretical X-ray data of medium or high resolution, both xIAM and xTAAM gave similarity indexes averaged over all non-H atoms close to zero (*S*_12_ = 0.1 or less), meaning there was almost no difference (Fig. 9 ▸, Table S3). However, for low resolution, the indexes were higher and there was a visible improvement of the ellipsoid shape with xTAAM compared with xIAM. The improvement was of similar magnitude for xSFth and xSFex.

**Figure 10:**
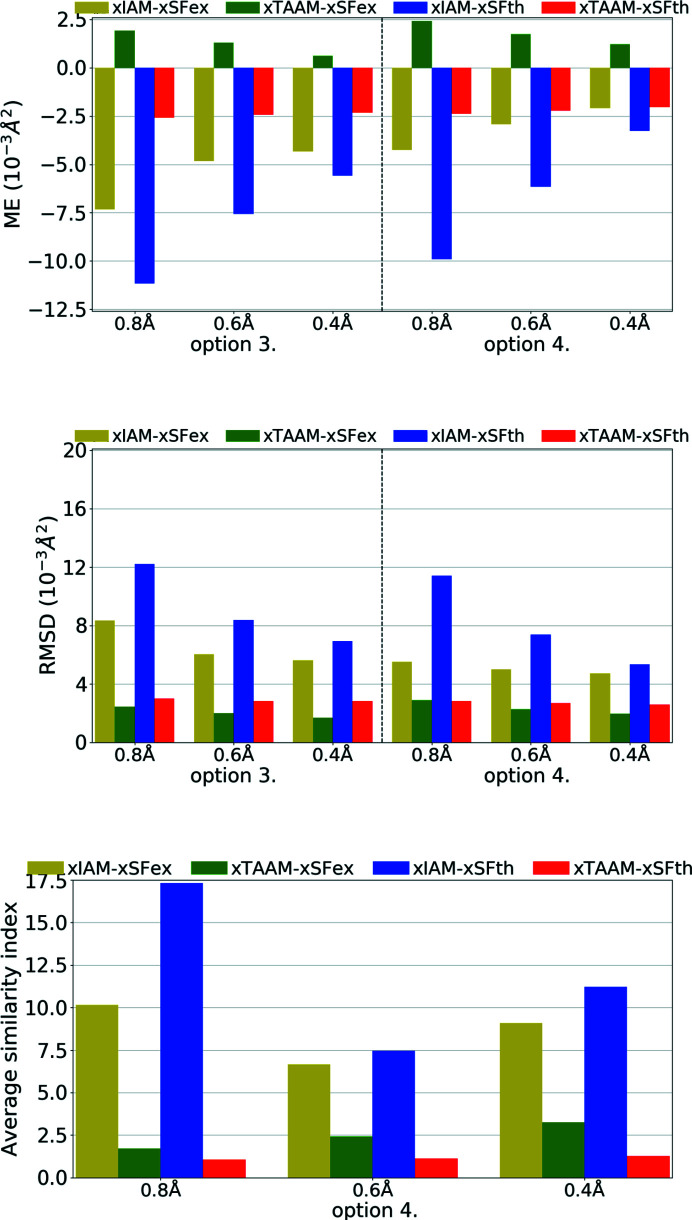
The mean error (ME) and the root-mean-square difference (RMSD) of *U*_iso_/*U*_eq_ (10^−3^ Å^2^) and the average similarity index of anisotropic ADPs for H atoms for structure refinements with experimental (xSFex) or theoretical (xSFth) X-ray diffraction data sets truncated to various resolutions (*d*_min_ = 0.8, 0.6, 0.4 Å) using various scattering models (xIAM, xTAAM) and sets of refined parameters (options 3 or 4). The indicators were computed with reference to values from neutron diffraction collected from the carbamazepine crystal.

The *U*_iso_’s for H atoms were, unexpectedly, slightly better modelled during refinement with experimental X-ray data sets than with the theoretical ones (Fig. 10 ▸, Table S3); however differences are smaller than mean e.s.d.s for the *U*_iso_/*U*_eq_ of H atoms (Table S3). The effect of lowering the error when xIAM was replaced by xTAAM was similar for both theoretical and experimental data. Interestingly, large improvements in H-atom *U*_iso_’s introduced by xTAAM when compared with xIAM were visible for all resolution ranges, by about 15–30% of the reference value, not only for low resolution as observed for non-H atoms.

Refinement of anisotropic ADPs for H atoms (option 4) with xIAM was difficult. Firstly, as mentioned above, with low-resolution X-ray data sets (both xSFth and xSFex), some H-atom ADPs went NPD. Strongly prolate or oblate thermal ellipsoids for the remaining H atoms were obtained, which resulted in large average similarity indexes. For the medium- and high-resolution data sets, the ADPs were still poorly shaped. In the case of xTAAM, refinements were stable for all resolutions and both the xSFth and xSFex data sets. Improved shapes for the H-atom displacement ellipsoids (smaller similarity indexes) were obtained. For experimental data the accuracy of the shapes decreased with better resolution for both xIAM and xTAAM. For the theoretical data, the accuracy seemed to depend much less on resolution for xTAAM. Nevertheless, the analysis confirms that, using theoretical X-ray data, it was possible to refine H-atom ADPs with xTAAM, similarly to the experimental X-ray data, and the accuracy of the shape of the obtained ADPs approached the accuracy for non-H-atom ADPs. Moreover, the improvement in accuracy when changing xIAM to xTAAM was comparable for theoretical and experimental data.

In summary, the main statistical trends were the same for xSFth and xSFex. Trends in *R*1 obtained during refinement of different models with xSFth and xSFex were the same. *R*1 decreased with fewer constraints and increased with increasing resolution for both xSFth and xSFex. The same is observed for the trends in maximum residuals. Residuals decreased with fewer constraints and increased with increasing resolution for both xSFth and xSFex. *X*—H bond lengths refined against xSFth were as close to those expected from neutron values as those refined against xSFex. The sizes and shapes of the ADPs of non-H atoms could be better modelled by refinement against xSFth (because these scattering factors were computed with neutron ADPs), but the trends are the same as for xSFex. For H atoms the trends in RMSD for *U*_eq_/*U*_iso_ were also maintained. It can be said that the quality of refinements against theoretical X-ray diffraction data largely matches that of good-quality experimental X-ray diffraction data. Thus we may expect that refinements against theoretical ED data will allow us to predict whether improvements in refinement statistics, geometry and thermal parameters will occur if the eIAM model is replaced by eTAAM.

#### 3.4.2. TAAM refinement with ED data   

Finally we present results showing the effects of the replacement of eIAM by eTAAM in crystal structure refinement with ED data. Because of a lack of experimental structure factors above 0.6 Å resolution, we were not able to test ‘high-resolution’ refinements. In such cases we marked missing results on the plots and in the tables with ‘Θ’. Also, some refinements led to NPD ADPs. Such refinements obviously give meaningless geometry, so we did not analyse them. They are marked on the plots and in the tables with ‘X’. To avoid generating too many refinement options, we refrained from introducing another one with constrained values of anisotropic ADPs. And, on top of that, in the case of experimental ED data there is a dynamic scattering effect present and not accounted for during our refinements and probably having the greatest influence on the quality of anisotropic ADPs. The kinematical treatment, the only available one at the moment for TAAM refinement, does not allow more accurate results to be obtained from experimental data.

**Figure 11:**
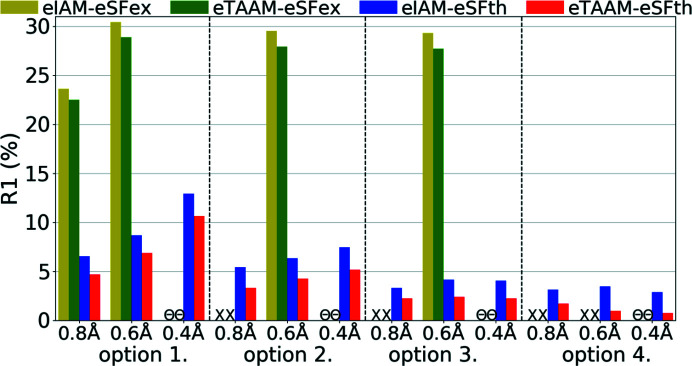
Overall *R*1 agreement factors (%) for structure refinements with experimental (eSFex) or theoretical (eSFth) ED data sets truncated to various resolutions (*d*_min_ = 0.8, 0.6, 0.4 Å) using various scattering models (eIAM, eTAAM) and sets of refined parameters (options 1, 2, 3 or 4).

In all analysed cases, the *R*1 factor was lower for the eTAAM refinement when compared with eIAM (Fig. 11 ▸, Table S3). The differences in *R*1 between eTAAM and eIAM for ED refinements were comparable with differences observed by X-ray diffraction. After introduction of eTAAM, the *R*1 decreased by 1.1–2.5% for theoretical, and 1.0–1.6% for experimental, ED data.

It must be noted, however, that in the case of experimental ED data the *R*1 values were much higher than for any other data set analysed here, ranging from 20% to 30%. Some systematic effects present only in these data, most likely dynamic scattering, were not taken into account during refinement. Nevertheless, improvements resulting from using a more accurate scattering model were visible in the *R*1 values and were of the same order of magnitude on an absolute scale as predicted by theoretical simulations.

Interestingly, the *R*1 values for theoretical ED data, when compared with X-ray, were more sensitive to restraints and constraints imposed on H-atom positions and thermal parameters. The *R*1 value did not change significantly for electron data when anisotropic ADPs for non-H atoms were allowed to be refined (transition from option 1 to option 2), and dropped noticeably when anisotropic ADPs for H atoms were allowed to be refined (from option 3 to 4).

The same phenomena occurred for refinements against ED data as for X-ray data. The best *R*1 values obtained with option 4 were smaller than the ones computed for the target geometry (Table 2 ▸). The scale of the differences was comparable with that from the xSFth refinements.

**Figure 12:**
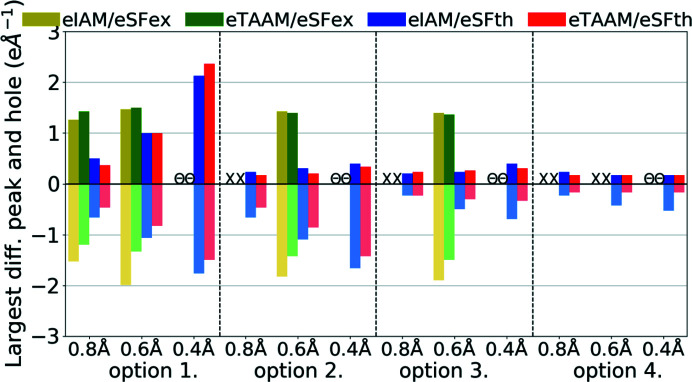
Largest difference peak and hole (e Å^−1^) on residual density Fourier maps for structure refinements with experimental (eSFex) or theoretical (eSFth) ED data sets truncated to various resolutions (*d*_min_ = 0.8, 0.6, 0.4 Å) using various scattering models (eIAM, eTAAM) and sets of refined parameters (options 1, 2, 3 or 4).

Analysis of residual electrostatic potential density, done on the basis of the largest differential peaks and holes, confirmed that eTAAM could be better fitted to the diffraction data than eIAM (Fig. 12 ▸, Table S3). In the case of ED, eTAAM led to smaller residual values, with negative values being usually much more reduced than positive values. For theoretical ED data, eTAAM improved residual density for all resolutions and all options of refined parameters, except for the specific combination of high-resolution data and option 1. Holes were reduced by up to 0.38 e Å^−1^ and the peaks up to 0.12 e Å^−1^. The values for the largest peaks and holes for experimental electron data were in general much larger than for theoretical, and did not decrease upon release of the positional and thermal parameter constraints. Nevertheless, improvements in the absolute difference due to the usage of eTAAM were similar in magnitude to those from the theoretical data.

Analysis of weighting parameters *a* and *b* (Table S3) is more difficult than in the case of X-ray structure factors (xSF), but we can conclude that values for eSFth are reasonable (*a* usually below 0.100, *b* usually close to 0.000) while for eSFex they indicate problems with model refinements (for eIAM weighting parameters due to too large discrepancies between the model and the data were not optimized at all, while for eTAAM a very high *b* value was found).

**Figure 13:**
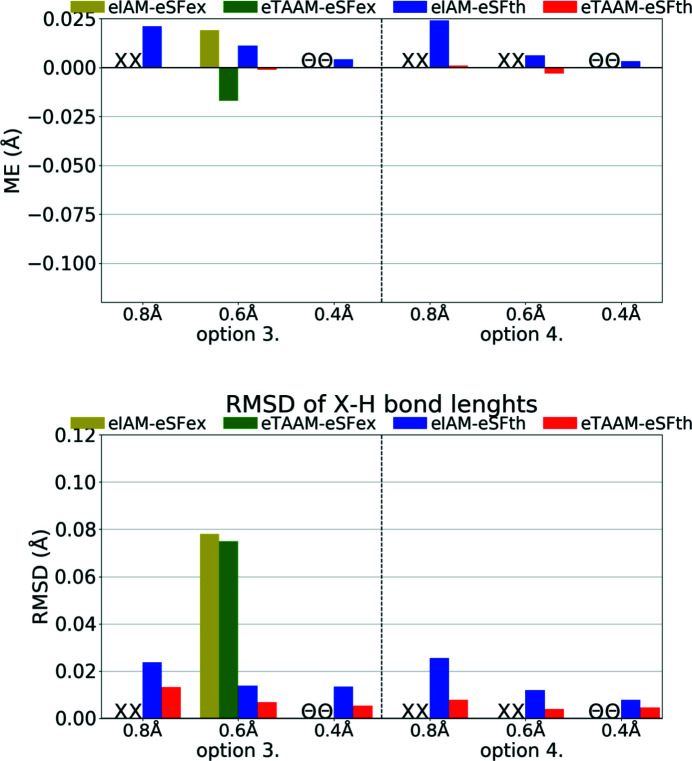
The mean error (ME) and the root-mean-square difference (RMSD) of the *X*—H bond lengths (Å) for structure refinements with experimental (eSFex) or theoretical (eSFth) ED data sets truncated to various resolutions (*d*_min_ = 0.8, 0.6, 0.4 Å) using various scattering models (eIAM, eTAAM) and sets of refined parameters (options 3 or 4). The ME was computed with reference to values from neutron diffraction collected from the carbamazepine crystal.

Another factor, perhaps even more important than fitting parameters, is the correctness of the obtained crystal structure (geometry, bond distances, quality of thermal parameters *etc*.). As was shown for X-ray data, xIAM leads to *X*—H bonds that are systematically too short while xTAAM refinement gives values closer to neutron values. Improvement occurs in the case of ED (Fig. 13 ▸, Table S3); however the problem then becomes different. For theoretical ED data it is clear that the accuracy of the *X*—H bonds determined with the use of the IAM model is already much better than it is for X-ray diffraction. Moreover, the eIAM refinements give *X*—H bonds that are slightly longer than the reference values from the neutron diffraction experiment (RMSD 0.013–0.024 Å). With eTAAM, the bond lengths were usually closer to neutron values, but there was some variance and they were often significantly either too short or too long. However, the improvement with use of eTAAM (RMSD 0.004–0.013 Å) is greater than the mean e.s.d.s of *X*—H bond lengths (Table S3).

For experimental ED data and eIAM, the *X*—H bonds were either too short or too long, there was no visible trend for either of these two possibilities and by using eTAAM the results did not become significantly closer to neutron reference values.

**Figure 14:**
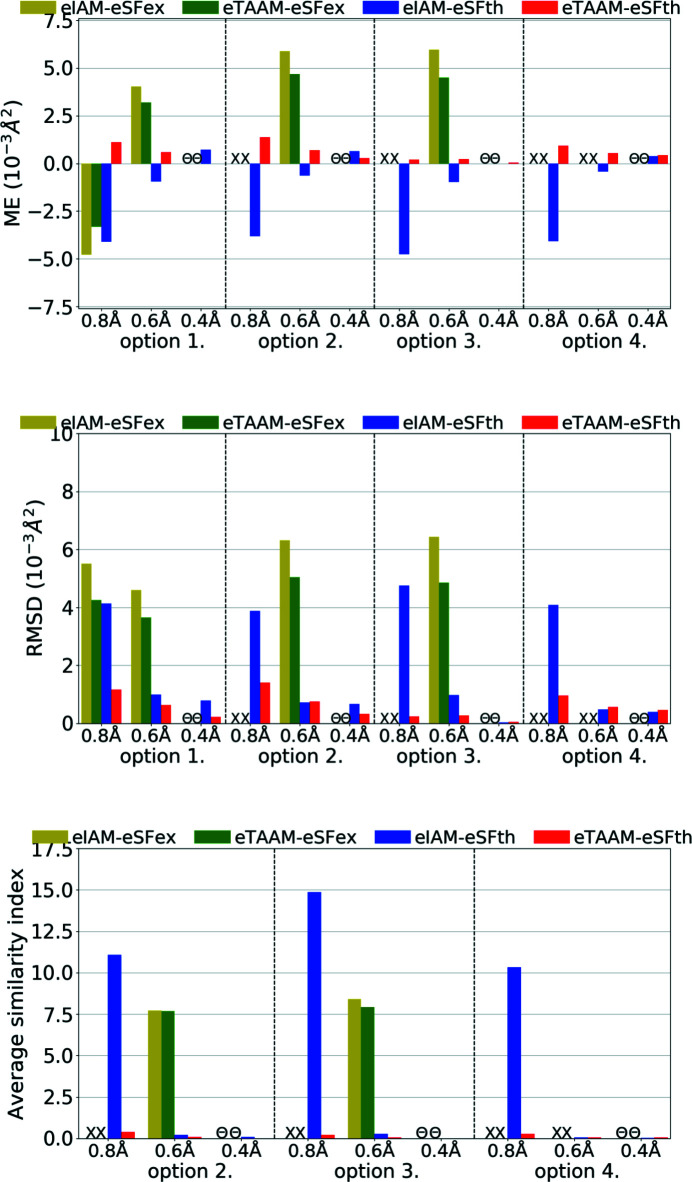
The mean error (ME) and the root-mean-square difference (RMSD) of *U*_iso_/*U*_eq_ (10^−3^ Å^2^), and the average similarity index of anisotropic ADPs for H atoms for structure refinements with experimental (eSFex) or theoretical (eSFth) ED data sets truncated to various resolutions (*d*_min_ = 0.8, 0.6, 0.4 Å) using various scattering models (eIAM, eTAAM) and sets of refined parameters (options 3 or 4). The indicators were computed with reference to values from neutron diffraction collected from the carbamazepine crystal.

The *U*_iso_/*U*_eq_ parameters, reflecting the size of ADPs, were far too small (by *ca* 37%) for non-H atoms for the eIAM refinements against theoretical ED data of low resolution (Fig. 14 ▸, Table S3). With eTAAM, they differentiated from the target values much less: they were too large by *ca* 9%. The effects of using eTAAM for medium and high resolution were smaller, but still significant (bigger than mean e.s.d.s. for *U*_iso_/*U*_eq_ parameters).

For experimental ED data with both eIAM and eTAAM, ADPs were too small in the case of low resolution and too large in the case of medium resolution. When anisotropic ADPs for non-H atoms were refined using options 2, 3 and 4, with low-resolution data all of them went NPD. When compared with theoretical simulations, the results for the medium-resolution set and eIAM appear suspicious as the ADPs are probably too high according to theoretical simulation. Nevertheless eTAAM improved the magnitude of ADPs, although improvement is on the limit of significance.

Concerning the shapes of anisotropic ADPs for non-H atoms, it seems that low-resolution ED data were very sensitive to the scattering model used. And this was true not only for experimental data but also for theoretical simulations. For theoretical data, the eIAM refinement with option 3 led to a NPD ADP for one atom, and with options 2 and 4 all ADPs were positive definite but had poor-quality shapes. The average similarity indexes were equal to 14.87 for option 3 (Fig. 14 ▸, Table S3). Refinements of the eIAM model against theoretical data of medium and high resolution resulted in much better shapes for ADPs, with the mean similarity indexes equal to about 0.2 for medium resolution, and below 0.1 for high resolution. The eTAAM model gave quite accurate values at low resolutions (0.22 for option 3) and equally good results at medium and high resolution.

For experimental ED data the improvements by eTAAM were not visible for low-resolution data. Both methods, eIAM and eTAAM, led to NPD ADPs for almost all non-H atoms. For medium resolution, the benefits of using eTAAM to estimate the shape of the ADPs were not indicated by the results. The best possible ADPs obtained from the currently available experimental ED data were of the wrong shape – they were flat or elongated. A better strategy for experimental measurement techniques, and more important dynamic scattering treatment during the refinement, may be required to obtain more accurate ADPs, and with current data and software availability it is still necessary to use some restraints while refining non-H-atom ADPs.

The simulations showed that trends in ADP behaviours for non-H atoms observed for ED data were analogous to those from X-ray data but occurred in the opposite direction. However, the error introduced by usage of the IAM model was larger for ED results than for X-ray, which was especially apparent at low resolution. Obtaining proper anisotropic ADPs from ED data of low resolution (*d*_min_ = 0.8 Å) with IAM is more difficult than from X-ray diffraction, in contrast to *X*—H bond lengths. This explains why researchers often report trouble with ADP refinements while analysing ED/scattering data (Wlodawer *et al.*, 2017 ▸). TAAM has the potential to solve this problem.

It must be noticed that this entire analysis is based on average values, which take into account 15 carbon atoms, two nitro­gen atoms and one oxygen atom whose contributions are not equal. As mentioned previously, good scattering factors are especially relevant to negatively charged atoms – such as oxygen. However, in our analysis trends for O1 with eIAM were the same as for average values. The difference for O1 was always smaller than the RMSD for all non-H atoms. Thus it cannot be confirmed that negatively charged oxygen atoms behave exceptionally, at least at the resolution ranges examined here.

**Figure 15:**
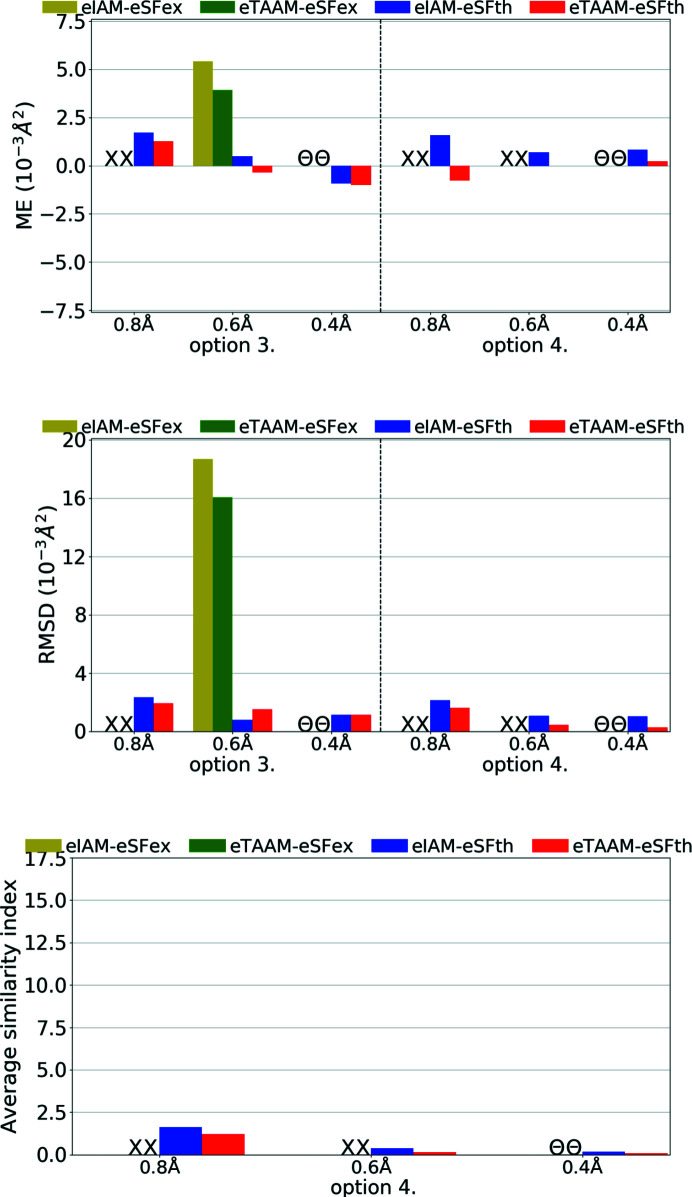
The mean error (ME) and the root-mean-square difference (RMSD) of *U*_iso_/*U*_eq_ (10^−3^ Å^2^), and the average similarity index of anisotropic ADPs for H atoms for structure refinements with experimental (eSFex) or theoretical (eSFth) ED data sets truncated to various resolutions (*d*_min_ = 0.8, 0.6, 0.4 Å) using various scattering models (eIAM, eTAAM) and sets of refined parameters (options 3 or 4). The indicators were computed with reference to values from neutron diffraction collected from the carbamazepine crystal.

For H atoms the accuracy of refined *U*_iso_/*U*_eq_ parameters with the use of eIAM was already very good in the case of theoretical electron data (Fig. 15 ▸, Table S3), comparable with the accuracy achieved for H atoms from X-ray diffraction when eTAAM was used. Introduction of eTAAM had little effect on the magnitudes of H-atom ADPs, even at low resolution, which was the opposite to what was observed for non-H atoms. Nevertheless, there was still an error that remained. The introduction of anisotropic ADPs for H atoms (option 4) does not change the results.

For experimental ED data the differences between refinements were smaller than the e.s.d.s of the *U*_iso_ of H atoms, as both successful refinements led to a much worse accuracy of the H-atom *U*_iso_ than theoretical simulation predicted could be achieved.

Improvements in the shape of H-atom ADPs can only be discussed for theoretical data. It must be noted that eIAM already gives a very good average similarity index for H-atom ADPs, as opposed to non-H atoms or H atoms from X-ray refinements. eTAAM improved further the accuracy but improvements were small.

## 4. Conclusions   

Although ED and cryo-EM methods have made enormous progress in recent years and an increasing number of atomic and near-atomic resolution structures are becoming available, interpretation of the data collected by these experiments continues to rely on an approximate scattering model based on spherical independent atoms, ignoring any charge redistributions due to chemical bonding.

Here we introduce the transferable aspherical atom model (TAAM) to electron crystallography. The model visibly improves model fitting statistics when compared with IAM and allows for reliable refinement of ADPs of non-H atoms – parameters that are the least well fit by IAM refinement methods. Other parameters (*e.g*. the *X*—H bond lengths, ADPs for H atoms) are also improved by TAAM. The improvements are even more pronounced with poorer-resolution diffraction data.

In particular, according to theoretical simulated data of 0.8 Å resolution one may expect:

(i) A decrease in the *R*1 factor by 1–2%.

(ii) Anisotropic ADPs of non-H atoms within 2% of neutron diffraction reference data with a similarity index of 0.3. The IAM does not obviously generate anisotropic ADPs for all atoms, the obtained ones are about 40% smaller than the reference ones and their similarity index is larger than 10.

(iii) Isotropic ADPs of H atoms which are *ca* 7% too large whereas for IAM they are *ca* 8% too large. Anisotropic ADPs of H atoms can also be obtained with an expected similarity index of 1.2 and a 6% reduction in size (1.6 and 7% for IAM).

(iv) Correction of H-atom positions to achieve an RMSD of 0.01 Å, although with IAM they are already quite good (RMSD = 0.02 Å).

It is expected that the improvements for TAAM over IAM will be even larger for data of resolution even lower than 0.8 Å. This however needs to be further investigated, not only for ED but also for single-particle cryo-EM data.

## Supplementary Material

Supplementary figures and tables. DOI: 10.1107/S2053273319015304/ae5072sup1.pdf

## Figures and Tables

**Figure 3:**
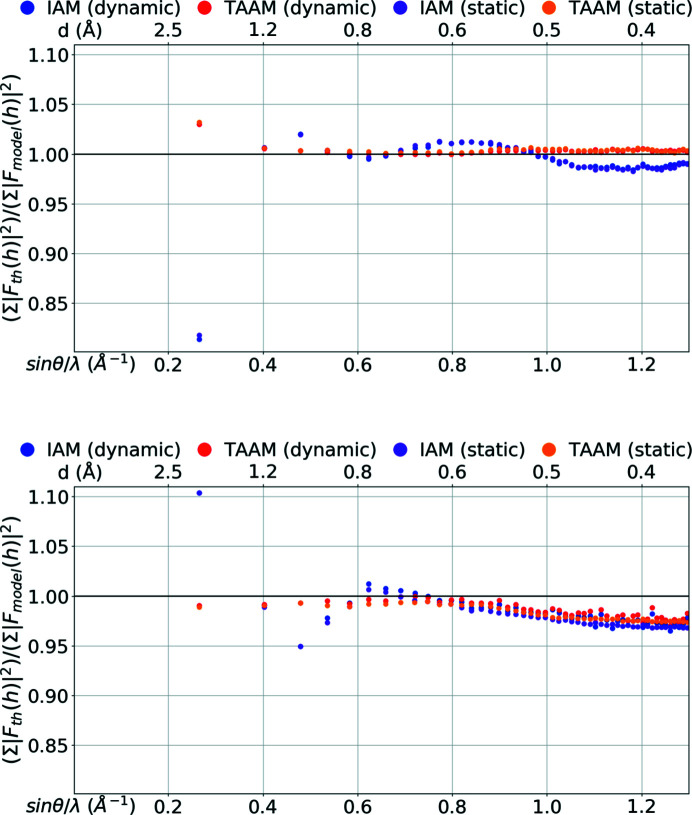
∑|*F*_th_(**h**)|^2^/ ∑|*F*_model_(**h**)|^2^ versus resolution [sin θ/λ (Å^−1^), *d* (Å)] for the IAM and TAAM models applied to the target crystal structure: (top) electron |*F*(**h**)| (Å), (bottom) X-ray |*F*(**h**)| (e). | *F*(**h**)| were computed using the target atomic positions and thermal parameters (dynamic) or with target atomic positions only (static). |*F*(**h**)| were divided into 50 equally populated bins over the range of resolutions.

**Table 1:** Refinement options summary A list of refined parameters for a particular option is given; in all cases the constrained/restrained parameters are indicated.

	Non-H atoms	H atoms
Option 1	*x*, *y*, *z*, *U*_iso_	*x*, *y*, *z*, *U*_iso_ – all constrained/restrained
Option 2	*x*, *y*, *z*, *U*_aniso_	*x*, *y*, *z*, *U*_iso_ – all constrained/restrained
Option 3	*x*, *y*, *z*, *U*_aniso_	*x*, *y*, *z*, *U*_iso_
Option 4	*x*, *y*, *z*, *U*_aniso_	*x*, *y*, *z*, *U*_aniso_

**Table 2:** The overall *R*1 factor (%) computed for the entire resolution range, *i.e* from *d*_max_ = 8.5 Å to *d*_min_ given in Table 2 ▸, for the IAM and TAAM models applied to the target crystal structure including: target atomic positions and thermal parameters (dynamic structure factors) or target atomic positions only (static structure factors) The *R*1 factor is defined as ∑||*F*_th_(**h**)| − |*F*_model_(**h**)|/∑|*F*_th_(**h**)|.

	Electron				X-ray		
*d*_min_ (Å)	0.83	0.60	0.38		0.83	0.60	0.38
Dynamic structure factors							
IAM	5.15	3.83	2.99		4.84	3.39	2.77
TAAM	1.23	1.01	0.89		1.01	1.04	1.49
Static structure factors							
IAM	4.92	3.44	2.45		4.89	3.09	2.07
TAAM	1.18	0.96	0.91		0.97	0.83	1.05

## References

[bb1] Allen, F. H. & Bruno, I. J. (2010). *Acta Cryst.* B**66**, 380–386. 10.1107/S0108768110012048 20484809

[bb2] Bak, J. M., Domagala, S., Hübschle, C., Jelsch, C., Dittrich, B. & Dominiak, P. M. (2011). *Acta Cryst.* A**67**, 141–153. 10.1107/S0108767310049731 21325717

[bb3] Bartesaghi, A., Matthies, D., Banerjee, S., Merk, A. & Subramaniam, S. (2014). *Proc. Natl Acad. Sci. USA*, **111**, 11709–11714. 10.1073/pnas.1402809111 PMC4136629 25071206

[bb4] Becke, A. D. (1988). *Phys. Rev. A*, **38**, 3098–3100. 10.1103/physreva.38.3098 9900728

[bb5] Bethe, H. (1930). *Ann. Phys.* **397**, 325–400.

[bb6] Binshtein, E. & Ohi, M. D. (2015). *Biochemistry*, **54**, 3133–3141. 10.1021/acs.biochem.5b00114 25955078

[bb7] Bojarowski, S. A., Kumar, P. & Dominiak, P. M. (2016). *ChemPhysChem*, **17**, 2455–2460. 10.1002/cphc.201600390 27166026

[bb8] Brown, A., Long, F., Nicholls, R. A., Toots, J., Emsley, P. & Murshudov, G. (2015). *Acta Cryst.* D**71**, 136–153. 10.1107/S1399004714021683 PMC4304694 25615868

[bb9] Carroni, M. & Saibil, H. R. (2016). *Methods*, **95**, 78–85. 10.1016/j.ymeth.2015.11.023 PMC5405050 26638773

[bb10] Cheng, Y. (2015). *Cell*, **161**, 450–457. 10.1016/j.cell.2015.03.049 PMC4409662 25910205

[bb11] Cheng, Y., Grigorieff, N., Penczek, P. A. & Walz, T. (2015). *Cell*, **161**, 438–449. 10.1016/j.cell.2015.03.050 PMC4409659 25910204

[bb12] Chodkiewicz, M. L., Migacz, S., Rudnicki, W., Makal, A., Kalinowski, J. A., Moriarty, N. W., Grosse-Kunstleve, R. W., Afonine, P. V., Adams, P. D. & Dominiak, P. M. (2018). *J. Appl. Cryst.* **51**, 193–199. 10.1107/S1600576717015825 PMC5822993 29507550

[bb13] Civalleri, B., Zicovich-Wilson, C. M., Valenzano, L. & Ugliengo, P. (2008). *CrystEngComm*, **10**, 405–410.

[bb14] Clabbers, M. T. B., Gruene, T., van Genderen, E. & Abrahams, J. P. (2019). *Acta Cryst.* A**75**, 82–93. 10.1107/S2053273318013918 PMC6302931 30575586

[bb15] Clabbers, M. T. B., van Genderen, E., Wan, W., Wiegers, E. L., Gruene, T. & Abrahams, J. P. (2017). *Acta Cryst.* D**73**, 738–748. 10.1107/S2059798317010348 PMC5586247 28876237

[bb16] Cowley, J. M., Peng, L. M., Ren, G., Dudarev, S. L. & Whelan, M. J. (2006). *International Tables for Crystallography*, Vol. C, edited by E. Prince, ch. 4.3.2. Chester: IUCr.

[bb17] Cruz, M. J. de la, Hattne, J., Shi, D., Seidler, P., Rodriguez, J., Reyes, F. E., Sawaya, M. R., Cascio, D., Weiss, S. C., Kim, S. K., Hinck, C. S., Hinck, A. P., Calero, G., Eisenberg, D. & Gonen, T. (2017). *Nat. Methods*, **14**, 399–402. 10.1038/nmeth.4178 PMC5376236 28192420

[bb18] Czyżnikowska, Z., Góra, R., Zaleśny, R., Lipkowski, P., Jarzembska, K., Dominiak, P. & Leszczynski, J. (2010). *J. Phys. Chem. B*, **114**, 9629–9644. 10.1021/jp101258q 20604521

[bb19] Dittrich, B., Hübschle, C. B., Luger, P. & Spackman, M. A. (2006). *Acta Cryst.* D**62**, 1325–1335. 10.1107/S090744490602899X 17057335

[bb20] Dittrich, B., Hübschle, C. B., Pröpper, K., Dietrich, F., Stolper, T. & Holstein, J. J. (2013). *Acta Cryst.* B**69**, 91–104. 10.1107/S2052519213002285 23719696

[bb21] Dolomanov, O. V., Bourhis, L. J., Gildea, R. J., Howard, J. A. K. & Puschmann, H. (2009). *J. Appl. Cryst.* **42**, 339–341.

[bb22] Domagala, S., Fournier, B., Liebschner, D., Guillot, B. & Jelsch, C. (2012). *Acta Cryst.* A**68**, 337–351. 10.1107/S0108767312008197 22514066

[bb23] Domagala, S. & Jelsch, C. (2008). *J. Appl. Cryst.* **41**, 1140–1149.

[bb24] Domagała, S., Munshi, P., Ahmed, M., Guillot, B. & Jelsch, C. (2011). *Acta Cryst.* B**67**, 63–78. 10.1107/S0108768110041996 21245542

[bb25] Dominiak, P. M. (2014). *Wiad. Chem.* **68**, 429–455.

[bb26] Dominiak, P. M., Volkov, A., Dominiak, A. P., Jarzembska, K. N. & Coppens, P. (2009). *Acta Cryst.* D**65**, 485–499. 10.1107/S0907444909009433 PMC2672818 19390154

[bb27] Dominiak, P. M., Volkov, A., Li, X., Messerschmidt, M. & Coppens, P. (2007). *J. Chem. Theory Comput.* **3**, 232–247. 10.1021/ct6001994 26627168

[bb28] Dovesi, R., Orlando, R., Erba, A., Zicovich-Wilson, C. M., Civalleri, B., Casassa, S., Maschio, L., Ferrabone, M., De La Pierre, M., D’Arco, P., Noël, Y., Causà, M., Rérat, M. & Kirtman, B. (2014). *Int. J. Quantum Chem.* **114**, 1287–1317.

[bb29] Dovesi, R., Saunders, V. R., Roetti, C., Orlando, R., Zicovich-Wilson, C. M., Pascale, F., Civalleri, B., Doll, K., Harrison, N. M., Bush, I. J., D’Arco, P., Llunell, M., Causà, M. & Noël, Y. (2016). *CRYSTAL14*. User’s Manual. University of Torino, Italy.

[bb30] Dubochet, J., Frank, J. & Henderson, R. (2017). *Nature*, **550**, 7675.

[bb31] Dudka, A. P., Avilov, A. S. & Lepeshov, G. G. (2008). *Crystallogr. Rep.* **53**, 530–536.

[bb32] Dunning, T. H. (1970). *J. Chem. Phys.* **53**, 2823–2833.

[bb33] Erba, A., Ferrabone, M., Orlando, R. & Dovesi, R. (2013). *J. Comput. Chem.* **34**, 346–354. 10.1002/jcc.23138 23081746

[bb34] Farrugia, L. J. (2012). *J. Appl. Cryst.* **45**, 849–854.

[bb35] Frisch, M. J., Trucks, G. W., Schlegel, H. B., Scuseria, G. E., Robb, M. A., Cheeseman, J. R., Scalmani, G., Barone, V., Mennucci, B., Petersson, G. A., Nakatsuji, H., Caricato, M., Li, X., Hratchian, H. P., Izmaylov, A. F., Bloino, J., Zheng, G., Sonnenberg, J. L. & Had, M. (2009). *Gaussian09.* Gaussian Inc., Wallingford, Connecticut, USA.

[bb36] Genderen, E. van, Clabbers, M. T. B., Das, P. P., Stewart, A., Nederlof, I., Barentsen, K. C., Portillo, Q., Pannu, N. S., Nicolopoulos, S., Gruene, T. & Abrahams, J. P. (2016). *Acta Cryst.* A**72**, 236–242. 10.1107/S2053273315022500 PMC4770873 26919375

[bb37] Grabowsky, S., Luger, P., Buschmann, J., Schneider, T., Schirmeister, T., Sobolev, A. N. & Jayatilaka, D. (2012). *Angew. Chem. Int. Ed.* **51**, 6776–6779. 10.1002/anie.201200745 22644673

[bb38] Grimme, S. (2006). *J. Comput. Chem.* **27**, 1787–1799. 10.1002/jcc.20495 16955487

[bb39] Groom, C. R., Bruno, I. J., Lightfoot, M. P. & Ward, S. C. (2016). *Acta Cryst.* B**72**, 171–179. 10.1107/S2052520616003954 PMC4822653 27048719

[bb40] Gruene, T., Wennmacher, J. T. C., Zaubitzer, C., Holstein, J. J., Heidler, J., Fecteau-Lefebvre, A., De Carlo, S., Müller, E., Goldie, K. N., Regeni, I., Li, T., Santiso-Quinones, G., Steinfeld, G., Handschin, S., van Genderen, E., van Bokhoven, J. A., Clever, G. H. & Pantelic, R. (2018). *Angew. Chem. Int. Ed.* **57**, 16313–16317. 10.1002/anie.201811318 PMC6468266 30325568

[bb41] Hansen, N. K. & Coppens, P. (1978). *Acta Cryst.* A**34**, 909–921.

[bb42] Hibbs, D. E., Howard, S. T., Huke, J. P. & Waller, M. P. (2005). *Phys. Chem. Chem. Phys.* **7**, 1772–1778. 10.1039/b416614k 19787937

[bb43] Hryc, C. F., Chen, D.-H., Afonine, P. V., Jakana, J., Wang, Z., Haase-Pettingell, C., Jiang, W., Adams, P. D., King, J. A., Schmid, M. F. & Chiu, W. (2017). *Proc. Natl Acad. Sci. USA*, **114**, 3103–3108. 10.1073/pnas.1621152114 PMC5373346 28270620

[bb44] Hübschle, C. B. & Dittrich, B. (2011). *J. Appl. Cryst.* **44**, 238–240. 10.1107/S0021889810042482 PMC3253731 22477783

[bb45] Jarzembska, K. N. & Dominiak, P. M. (2012). *Acta Cryst.* A**68**, 139–147. 10.1107/S0108767311042176 22186290

[bb46] Jayatilaka, D. & Dittrich, B. (2008). *Acta Cryst.* A**64**, 383–393. 10.1107/S0108767308005709 18421128

[bb47] Jayatilaka, D. & Grimwood, D. J. (2001). *Acta Cryst.* A**57**, 76–86. 10.1107/s0108767300013155 11124506

[bb48] Jones, C. G., Martynowycz, M. W., Hattne, J., Fulton, T. J., Stoltz, B. M., Rodriguez, J. A., Nelson, H. M. & Gonen, T. (2018). *ACS Cent. Sci.* **4**, 1587–1592. 10.1021/acscentsci.8b00760 PMC6276044 30555912

[bb49] Koritsanszky, T., Howard, S., Richter, T., Su, Z., Mallinson, P. R. & Hansen, N. K. (2003). *XD: a computer program package for multipole refinement and analysis of electron densities from diffraction data*.

[bb50] Koritsanszky, T., Volkov, A. & Coppens, P. (2002). *Acta Cryst.* A**58**, 464–472. 10.1107/s0108767302010991 12192120

[bb51] Krishnan, R., Binkley, J. S., Seeger, R. & Pople, J. A. (1980). *J. Chem. Phys.* **72**, 650–654.

[bb52] Krysiak, Y., Barton, B., Marler, B., Neder, R. B. & Kolb, U. (2018). *Acta Cryst.* A**74**, 93–101. 10.1107/S2053273317018277 29493538

[bb53] Kulik, M., Goral, A. M., Jasiński, M., Dominiak, P. M. & Trylska, J. (2015). *Biophys. J.* **108**, 655–665. 10.1016/j.bpj.2014.12.020 PMC4317553 25650932

[bb54] Kumar, P., Bojarowski, S. A., Jarzembska, K. N., Domagała, S., Vanommeslaeghe, K., MacKerell, A. D. & Dominiak, P. M. (2014). *J. Chem. Theory Comput.* **10**, 1652–1664. 10.1021/ct4011129 PMC3985931 24803869

[bb55] Kumar, P., Cabaj, M. K., Pazio, A. & Dominiak, P. M. (2018). *IUCrJ*, **5**, 449–469. 10.1107/S2052252518006346 PMC6038959 30002846

[bb56] Kumar, P., Gruza, B., Bojarowski, S. A. & Dominiak, P. M. (2019). *Acta Cryst.* A**75**, 398–408. 10.1107/S2053273319000482 30821272

[bb57] Lee, C., Yang, W. & Parr, R. G. (1988). *Phys. Rev. B*, **37**, 785–789. 10.1103/physrevb.37.785 9944570

[bb58] Malinska, M. & Dauter, Z. (2016). *Acta Cryst.* D**72**, 770–779. 10.1107/S2059798316006355 PMC4908868 27303797

[bb59] Malinska, M., Jarzembska, K. N., Goral, A. M., Kutner, A., Wozniak, K. & Dominiak, P. M. (2014). *Acta Cryst.* D**70**, 1257–1270. 10.1107/S1399004714002351 24816095

[bb60] Merk, A., Bartesaghi, A., Banerjee, S., Falconieri, V., Rao, P., Davis, M. I., Pragani, R., Boxer, M. B., Earl, L. A., Milne, J. L. S. & Subramaniam, S. (2016). *Cell*, **165**, 1698–1707. 10.1016/j.cell.2016.05.040 PMC4931924 27238019

[bb61] Meyer, B., Guillot, B., Ruiz-Lopez, M. F. & Genoni, A. (2016). *J. Chem. Theory Comput.* **12**, 1052–1067. 10.1021/acs.jctc.5b01007 26799516

[bb62] Mott, F. L. (1930). *J. Bull.* **7**, 88.

[bb63] Nannenga, B. L., Shi, D., Leslie, A. G. W. & Gonen, T. (2014). *Nat. Methods*, **11**, 927–930. 10.1038/nmeth.3043 PMC4149488 25086503

[bb64] Nassour, A., Domagala, S., Guillot, B., Leduc, T., Lecomte, C. & Jelsch, C. (2017). *Acta Cryst.* B**73**, 610–625. 10.1107/S2052520617008204 28762971

[bb65] Palatinus, L., Brázda, P., Boullay, P., Perez, O., Klementová, M., Petit, S., Eigner, V., Zaarour, M. & Mintova, S. (2017). *Science*, **355**, 166–169. 10.1126/science.aak9652 28082587

[bb66] Palatinus, L., Corrêa, C. A., Steciuk, G., Jacob, D., Roussel, P., Boullay, P., Klementová, M., Gemmi, M., Kopeček, J., Domeneghetti, M. C., Cámara, F. & Petříček, V. (2015). *Acta Cryst.* B**71**, 740–751. 10.1107/S2052520615017023 26634732

[bb67] Palatinus, L., Petrícek, V. & Corrêa, C. A. (2015). *Acta Cryst.* A**71**, 235–244. 10.1107/S2053273315001266 25727873

[bb68] Pascale, F., Zicovich-Wilson, C. M., López Gejo, F., Civalleri, B., Orlando, R. & Dovesi, R. (2004). *J. Comput. Chem.* **25**, 888–897. 10.1002/jcc.20019 15011261

[bb69] Peng, L.-M. (1999). *Micron*, **30**, 625–648.

[bb70] Perdew, J. P. (1986). *Phys. Rev. B*, **33**, 8822–8824. 10.1103/physrevb.33.8822 9938299

[bb71] Petrícek, V., Dusek, M. & Palatinus, L. (2014). *Z. Kristallogr. Cryst. Mater.* **229**, 345–352.

[bb72] Pichon-Pesme, V., Lecomte, C. & Lachekar, H. (1995). *J. Phys. Chem.* **99**, 6242–6250.

[bb73] Sawaya, M. R., Rodriguez, J., Cascio, D., Collazo, M. J., Shi, D., Reyes, F. E., Hattne, J., Gonen, T. & Eisenberg, D. S. (2016). *Proc. Natl Acad. Sci. USA*, **113**, 11232–11236. 10.1073/pnas.1606287113 PMC5056061 27647903

[bb74] Sheldrick, G. M. (2015). *Acta Cryst.* A**71**, 3–8.

[bb75] Shi, D., Nannenga, B. L., Iadanza, M. G. & Gonen, T. (2013). *eLife*, **2**, e01345. 10.7554/eLife.01345 PMC3831942 24252878

[bb76] Smaalen, S. van, Palatinus, L. & Schneider, M. (2003). *Acta Cryst.* A**59**, 459–469. 10.1107/S010876730301434X 12944610

[bb77] Sovago, I., Gutmann, M. J., Senn, H. M., Thomas, L. H., Wilson, C. C. & Farrugia, L. J. (2016). *Acta Cryst.* B**72**, 39–50. 10.1107/S2052520615019538 26830795

[bb78] Spek, A. L. (2003). *J. Appl. Cryst.* **36**, 7–13.

[bb79] Subramaniam, S., Kühlbrandt, W. & Henderson, R. (2016). *IUCrJ*, **3**, 3–7. 10.1107/S2052252515023738 PMC4704073 26870375

[bb80] Volkov, A., King, H. F. & Coppens, P. (2006). *J. Chem. Theory Comput.* **2**, 81–89. 10.1021/ct050216x 26626382

[bb81] Volkov, A., Koritsanszky, T. & Coppens, P. (2004). *Chem. Phys. Lett.* **391**, 170–175.

[bb82] Volkov, A., Li, X., Koritsanszky, T. & Coppens, P. (2004). *J. Phys. Chem. A*, **108**, 4283–4300.

[bb83] Volkov, A., Macchi, P., Farrugia, L., Gatti, C., Mallinson, P., Richter, T. & Koritsanszky, T. (2016). *XD2016 - A Computer Program Package for Multipole Refinement, Topological Analysis of Charge Densities and Evaluation of Intermolecular Energies from Experimental and Theoretical Structure Factors.*

[bb84] Whitten, A. E. & Spackman, M. A. (2006). *Acta Cryst.* B**62**, 875–888. 10.1107/S0108768106020787 16983168

[bb85] Wilson, A. J. C. & Geist, V. (1993). *Cryst. Res. Technol.* **28**, 110.

[bb86] Wlodawer, A., Li, M. & Dauter, Z. (2017). *Structure*, **25**, 1589–1597.e1. 10.1016/j.str.2017.07.012 PMC5657611 28867613

[bb87] Woińska, M., Grabowsky, S., Dominiak, P. M., Woźniak, K. & Jayatilaka, D. (2016). *Sci. Adv.* **2**, e1600192. 10.1126/sciadv.1600192 PMC4928899 27386545

[bb88] Woińska, M., Jayatilaka, D., Dittrich, B., Flaig, R., Luger, P., Woźniak, K., Dominiak, P. M. & Grabowsky, S. (2017). *ChemPhysChem*, **18**, 3334–3351. 10.1002/cphc.201700810 29168318

[bb89] Yang, J. & Waller, M. P. (2013). *J. Comput. Chem.* **34**, 466–470. 10.1002/jcc.23155 23109274

[bb90] Yonekura, K., Kato, K., Ogasawara, M., Tomita, M. & Toyoshima, C. (2015). *Proc. Natl Acad. Sci.* **112**, 3368–3373. 10.1073/pnas.1500724112 PMC4372003 25730881

[bb91] Yonekura, K. & Maki-Yonekura, S. (2016). *J. Appl. Cryst.* **49**, 1517–1523.

[bb92] Zarychta, B., Pichon-Pesme, V., Guillot, B., Lecomte, C. & Jelsch, C. (2007). *Acta Cryst.* A**63**, 108–125. 10.1107/S0108767306053748 17301471

[bb93] Zheng, J.-C., Wu, L. & Zhu, Y. (2009). *J. Appl. Cryst.* **42**, 1043–1053.

[bb94] Zheng, J.-C., Zhu, Y., Wu, L. & Davenport, J. W. (2005). *J. Appl. Cryst.* **38**, 648–656.

[bb95] Zhong, S., Dadarlat, V. M., Glaeser, R. M., Head-Gordon, T. & Downing, K. H. (2002). *Acta Cryst.* A**58**, 162–170. 10.1107/s0108767301020256 11832586

