# Supplementary material for: Refinement of organic crystal structures with multipolar electron scattering factors
Source: Acta Crystallogr A Found Adv. 2020 Jan 1;76(Pt 1):92–109. doi: 10.1107/S2053273319015304 (PMC8127334; doi:10.1107/S2053273319015304)
Supplement: Supplementary file 1 [file a-76-00092-sup1.pdf]

FOUNDATIONS
ADVANCES

Volume 76 (2020)

Supporting information for article:

Refinement of organic crystal structure with multipolar electron scattering factors

Barbara Gruza, Michał Leszek Chodkiewicz, Joanna Krzeszczakowska and Paulina Maria Dominiak

Refinement of organic crystal structure with multipolar electron scattering factors

Barbara Gruza,[1] Michał Leszek Chodkiewicz,[1] Joanna Krzeszczakowska,[1] and Paulina Maria Dominiak[1]*

[1]*Biological and Chemical Research Centre, Department of Chemistry, University of Warsaw, ul. Żwirki i Wigury 101, 02-089, Warszawa, Poland*

Table S1. Unit cell dimensions from various methods.

	a (Å)	b (Å)	c (Å)	β (º)	V (Å³)
Theory (Crystal14/B3LYP/DZP), 0 K	7.4219	11.0423	13.5060	92.970	1105.78
Neutron diffraction, 100 K	7.4980(15)	11.058(2)	13.789(3)	92.838(16)	1141.9(4)
X-ray diffraction, 100 K	7.4893(4)	11.0323(5)	13.7640(6)	92.953(2)	1135.73(9)
Electron diffraction, 100 K	7.460(2)	11.040(2)	13.760(3)	92.61(3)	1132.1(4)

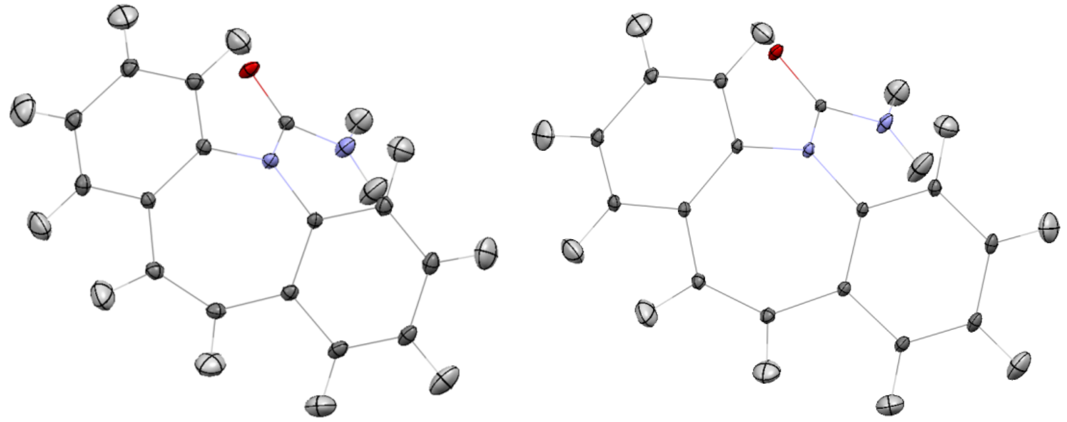

Fig. S1: The atomic displacement ellipsoids drawn at the 50% probability level from neutron diffraction (left) and from periodic quantum calculations (right) at Crystal14/B3LYP-D*/DZP level.

Table S2. Refinement statistics for theoretical electron diffraction data refined with option 2 in which the X-H bond lengths were constrained to values optimal for X-ray radiation. X indicates that results are missing due to problems with refinements (most ADPs were non-positive definite).

Resolution	Option	Model	R1[%]	peak/hole [eÅ^{-1}]	Overall scaling factor x100	WGHT a	WGHT b
0.83 Å	2	IAM	X	X	X	X	X
		TAAM	7.0	-0.26/0.14	100.53	0.11	0.24
0.6 Å	2	IAM	11.7	-0.54/0.24	99.34	0.20	0.00
		TAAM	8.6	-0.48/0.20	100.81	0.13	0.06
0.38 Å	2	IAM	11.1	-0.80/0.30	99.28	0.20	0.00
		TAAM	9.2	-0.74/0.30	98.90	0.07	0.01

Resolution	Option	Model	RMSD Ueq non-H [Å²]	RMSD Ueq non-H [%]	ME Ueq non-H [Å²]	Average similarity index Uaniso non-H
0.83 Å	2	IAM	X	X	X	X
		TAAM	0.00062	5.6	0.00058	3.53
0.6 Å	2	IAM	0.00160	14.4	0.00158	0.99
		TAAM	0.00105	9.5	0.00100	0.20
0.38 Å	2	IAM	0.00068	6.1	0.00068	0.09
		TAAM	0.00036	3.2	0.00023	0.02

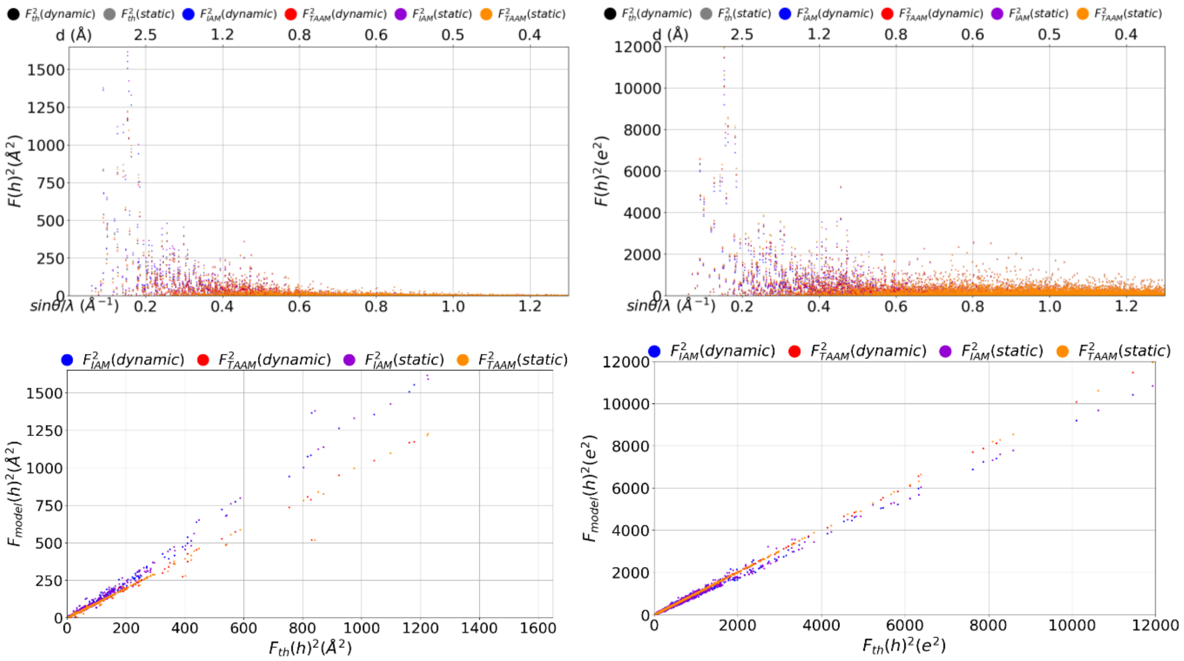

Fig S2. (top) $|F_{th}(\mathbf{h})|^2$ and $|F_{model}(\mathbf{h})|^2$ versus resolution (sinθ/λ ($Å^{-1}$), d (Å)) and (bottom) $|F_{model}(\mathbf{h})|^2$ versus $|F_{th}(\mathbf{h})|^2$ where model stands for IAM or TAAM applied to the target crystal structure: (left) electron $|F(\mathbf{h})|$ (Å), (right) x-ray $|F(\mathbf{h})|$ (e). $F(\mathbf{h})|$ were computed with target atomic positions and thermal parameters (dynamic) or with target atomic positions only (static).

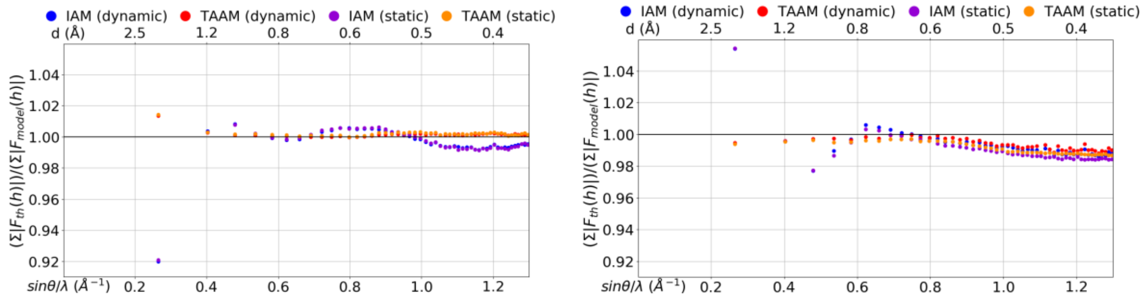

Fig S3. $\sum|F_{th}(\mathbf{h})|/\sum|F_{model}(\mathbf{h})|$ versus resolution (sinθ/λ ($Å^{-1}$), d (Å)) for IAM and TAAM models applied to the target crystal structure: (left) electron $|F(\mathbf{h})|$ (Å), (right) x-ray $|F(\mathbf{h})|$ (e). || $F(\mathbf{h})|$ was computed with target atomic positions and thermal parameters (dynamic) or with target atomic positions only (static). $|F(\mathbf{h})|$ were divided into 50 equally populated bins over the resolution range.

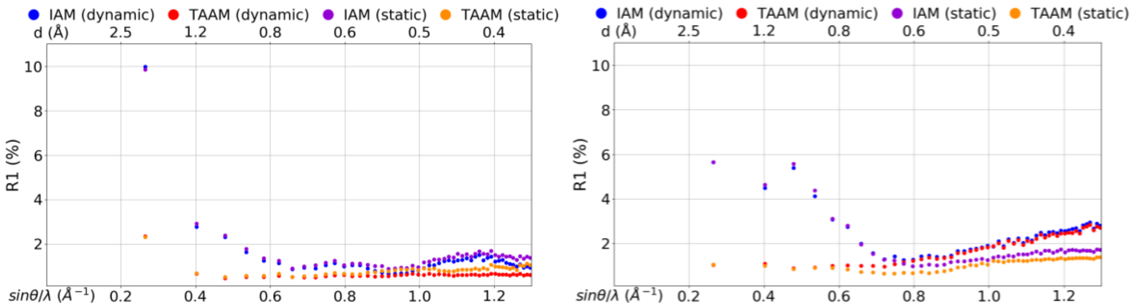

Fig S4. The R1 factors (%) versus resolution (sinθ/λ ($Å^{-1}$), d (Å)) for the IAM and TAAM models applied to the target crystal structure: (left) electron $|F(\mathbf{h})|$, (right) x-ray $|F(\mathbf{h})|$. | $F(\mathbf{h})|$ were computed with target atomic positions and thermal parameters (dynamic) or with target atomic positions only (static). $|F(\mathbf{h})|$ were divided into 50 equally populated bins over the resolution range. The R1 factor is defined as R1=$\sum||F_{th}(\mathbf{h})| - |F_{model}(\mathbf{h})|/\sum|F_{th}(\mathbf{h})|$.

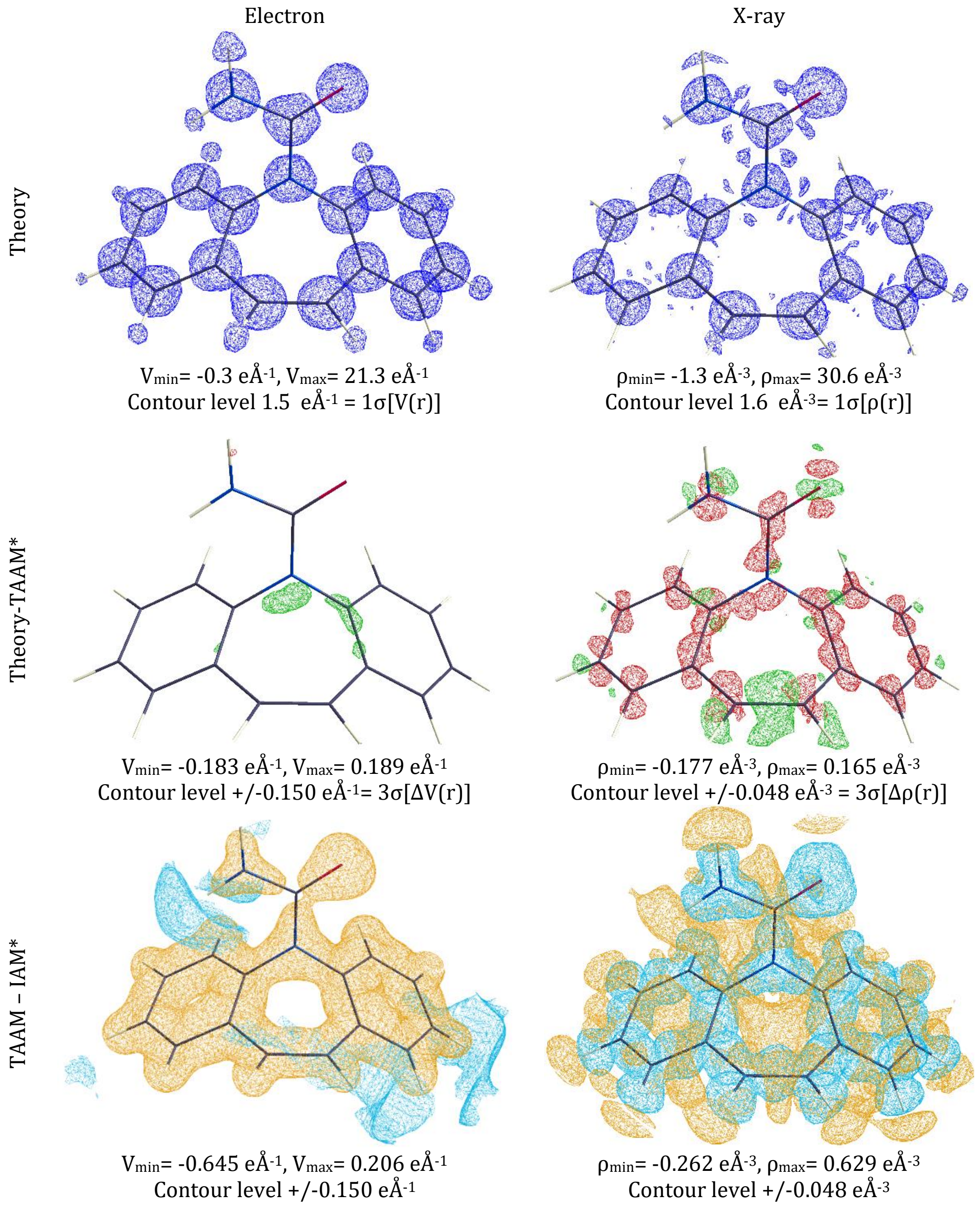

Fig. S5. Fourier density maps computed from the electron (eÅ^{-1}) and X-ray (eÅ^{-3}) dynamic structure factors of the carbamazepine crystal, truncated at d_{min} = 0.60 Å. Structure factors were computed for target atomic positions and thermal parameters. Theory - the $|F_{th}(\mathbf{h})|e^{i\varphi_{TAAM}}$ map; Theory–TAAM - for the $[|F_{th}(\mathbf{h})| - |F_{TAAM}(\mathbf{h})|]e^{i\varphi_{TAAM}}$ map the green contour indicates positive and the red negative values; TAAM–IAM - for the $|F_{TAAM}(\mathbf{h})|e^{i\varphi_{TAAM}} - |F_{IAM}(\mathbf{h})|e^{i\varphi_{IAM}}$ map the blue contour indicates positive and the orange negative values.

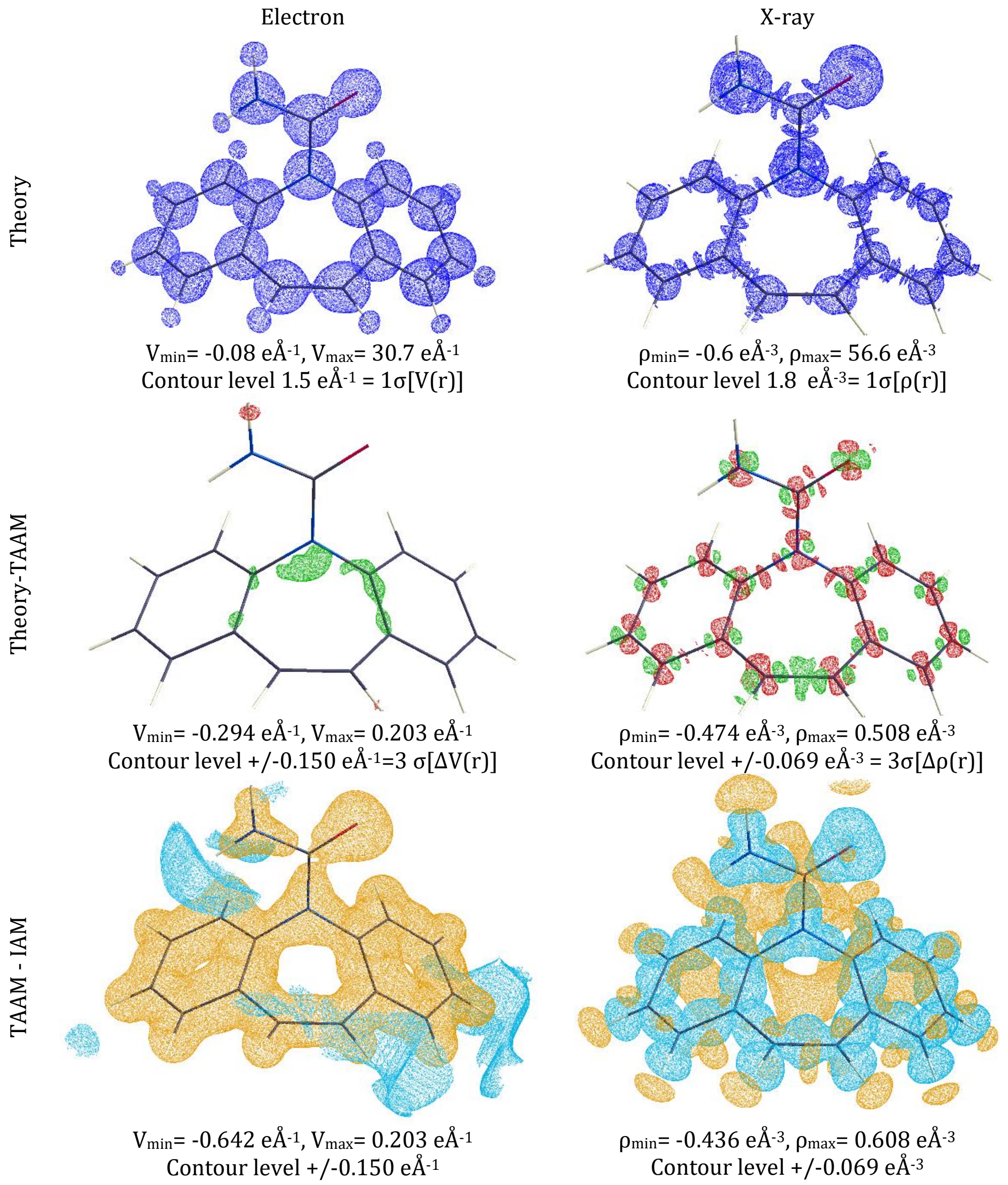

Fig. S6. Fourier density maps computed from the electron (eÅ^{-1}) and X-ray (eÅ^{-3}) dynamic structure factors of the carbamazepine crystal, truncated at d_{min} = 0.38 Å. Structure factors were computed for target atomic positions and thermal parameters. Theory - the $|F_{th}(\mathbf{h})|e^{i\varphi_{TAAM}}$ map; Theory–TAAM - for the $[|F_{th}(\mathbf{h})| - |F_{TAAM}(\mathbf{h})|]e^{i\varphi_{TAAM}}$ map the green contour indicates positive and the red negative values; TAAM–IAM - for the $|F_{TAAM}(\mathbf{h})|e^{i\varphi_{TAAM}} - |F_{IAM}(\mathbf{h})|e^{i\varphi_{IAM}}$ map the blue contour indicates positive and the orange negative values.

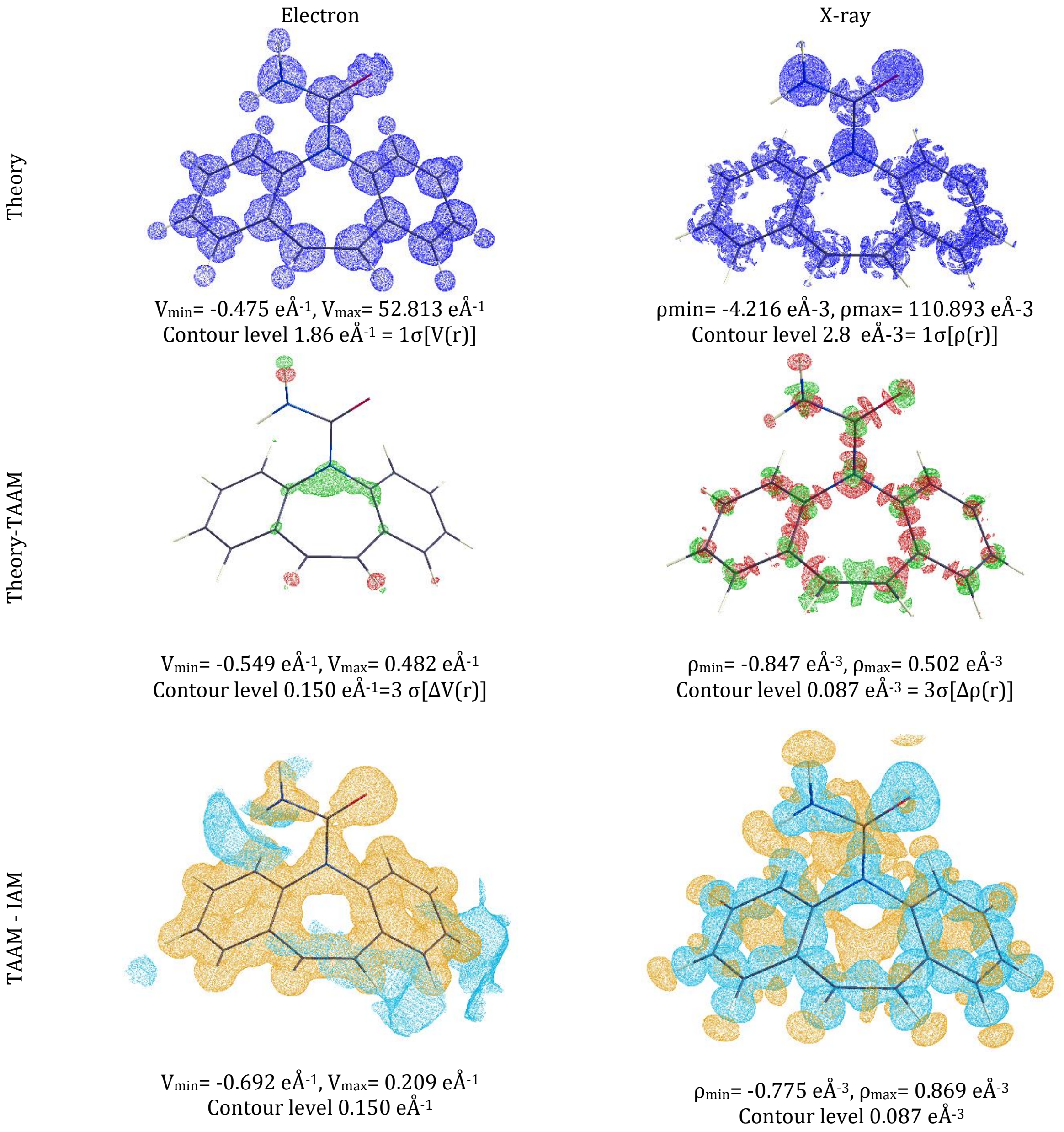

Fig. S7. Fourier density maps computed from the electron (eÅ^{-1}) and X-ray (eÅ^{-3}) static structure factors of the carbamazepine crystal, truncated at d_{min} = 0.38 Å. Structure factors were computed for target atomic positions and thermal parameters. Theory - the $|F_{th}(\mathbf{h})|e^{i\varphi_{TAAM}}$ map; Theory–TAAM - for the $[|F_{th}(\mathbf{h})| - |F_{TAAM}(\mathbf{h})|]e^{i\varphi_{TAAM}}$ map the green contour indicates positive and the red negative values; TAAM–IAM - for the $|F_{TAAM}(\mathbf{h})|e^{i\varphi_{TAAM}} - |F_{IAM}(\mathbf{h})|e^{i\varphi_{IAM}}$ map the blue contour indicates positive and the orange negative values.

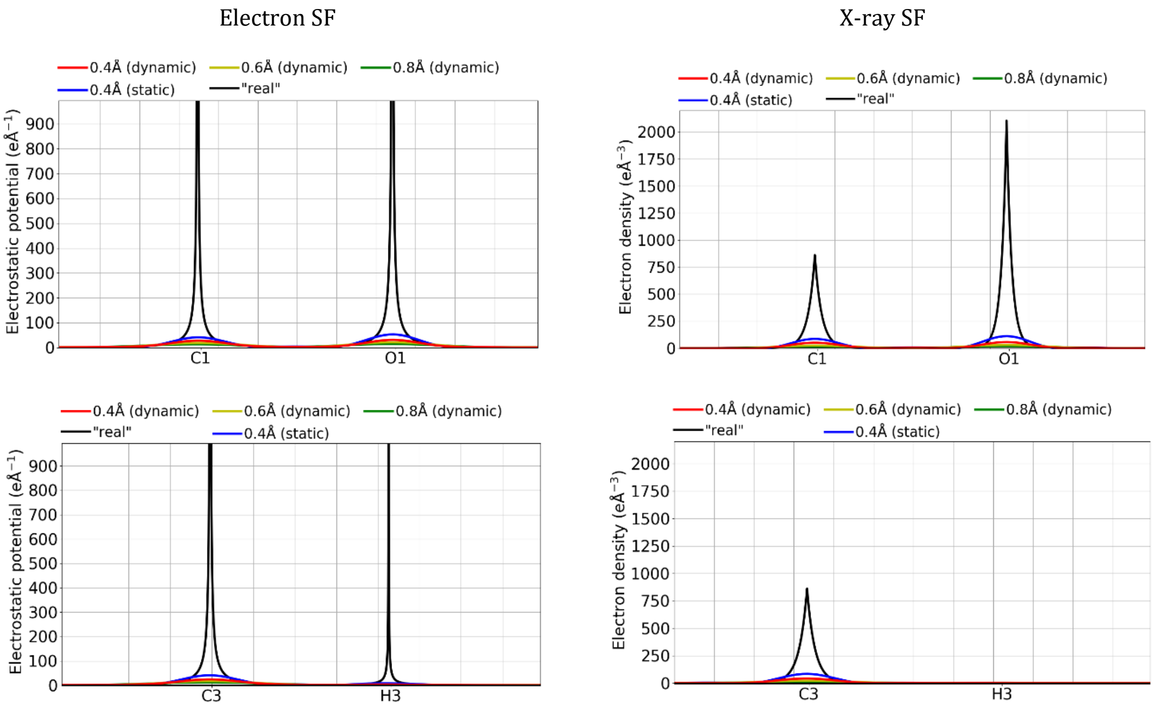

Fig. S8. Fourier density maps ($|F_{th}(\mathbf{h})|e^{i\varphi_{TAAM}}$) of electrostatic potential (eÅ^{-1}) or electron density (eÅ^{-3}) along selected covalent bonds computed from electron and X-ray dynamic and static structure factors from the carbamazepine crystal, truncated at various resolutions (Å). "Real" values computed directly from TAAM models without Fourier transformations are given for the reference. Values were computed for target atomic positions and thermal parameters.

Tables S3. Numerical values for results presented in Figures 6 – 16. Θ indicates that results are missing due to lack of structure factor data, X indicates that results are missing due to problems with refinements (NPD ADPs), – indicates that refinement was not performed. *0.42 Å for xSFex.

			R1 [%]							
Resolution	Option	Model	xSFth	xSFex	eSFth	eSFex	xSFth	xSFex	eSFth	eSFex
			a,b-fitted				a,b = 0			
0.83 Å	1	IAM	5.4	5.8	6.6	23.6	6.0	6.0	8.7	23.8
		TAAM	4.5	4.5	4.7	22.5	4.8	4.7	9.3	22.2
	2	IAM	3.0	3.6	5.4	X	4.4	3.7	X	X
		TAAM	2.3	2.7	3.3	X	3.2	2.7	9.5	X
	3	IAM	2.8	3.5	3.3	X	4.0	3.5	X	X
		TAAM	0.7	1.5	2.2	X	1.2	1.5	8.1	X
	4	IAM	2.7	3.4	3.1	X	X	–	–	–
		TAAM	0.6	1.4	1.7	X	1.1	–	–	–
0.6 Å	1	IAM	7.4	7.7	8.7	30.4	8.0	8.0	13.9	23.0
		TAAM	6.7	6.6	6.9	28.9	7.0	6.8	12.2	21.4
	2	IAM	3.0	4.2	6.4	29.5	5.7	4.3	X	X
		TAAM	2.0	2.8	4.3	27.9	3.3	2.8	12.4	X
	3	IAM	2.8	4.2	4.1	29.3	5.3	4.2	X	X
		TAAM	0.6	2.0	2.4	27.7	1.3	2.0	10.2	X
	4	IAM	2.8	4.2	3.5	X	X	–	–	–
		TAAM	0.5	2.0	1.0	X	1.2	–	–	–
0.38* Å	1	IAM	12.2	11.7	12.9	Θ	13.2	12.2	28.2	Θ
		TAAM	11.9	10.9	10.6	Θ	12.2	11.4	17.4	Θ
	2	IAM	1.9	5.5	7.4	Θ	7.7	5.5	X	Θ
		TAAM	1.4	4.4	5.1	Θ	4.0	4.4	18.3	Θ
	3	IAM	1.8	5.4	4.1	Θ	7.1	5.4	X	Θ
		TAAM	0.6	4.0	2.2	Θ	1.7	4.0	14.8	Θ
	4	IAM	1.8	5.4	2.9	Θ	X	5.4	–	Θ
		TAAM	0.5	4.0	0.7	Θ	1.6	4.0	–	Θ

Largest diff. peak and hole [xSF: eÅ^{-3}, eSF: eÅ^{-1}]

Resolution	Option	Model	xSFth		xSFex		eSFth		eSFex	
					a,b-fitted					
0.83 Å	1	IAM	-0.50	0.62	-0.42	0.60	-0.67	0.48	-1.54	1.27
		TAAM	-0.50	0.61	-0.45	0.43	-0.47	0.37	-1.19	1.43
	2	IAM	-0.20	0.21	-0.23	0.19	-0.66	0.22	X	X
		TAAM	-0.24	0.26	-0.21	0.24	-0.46	0.18	X	X
	3	IAM	-0.18	0.20	-0.23	0.17	-0.24	0.21	X	X
		TAAM	-0.05	0.11	-0.11	0.10	-0.23	0.22	X	X
	4	IAM	-0.19	0.19	-0.23	0.16	-0.23	0.22	X	X
		TAAM	-0.05	0.11	-0.11	0.09	-0.17	0.17	X	X
0.6 Å	1	IAM	-1.06	1.73	-0.78	1.47	-1.05	1.00	-1.99	1.48
		TAAM	-1.12	1.68	-0.92	1.31	-0.85	0.99	-1.33	1.50
	2	IAM	-0.22	0.53	-0.27	0.65	-1.08	0.29	-1.82	1.41
		TAAM	-0.44	0.34	-0.38	0.28	-0.88	0.20	-1.43	1.41
	3	IAM	-0.19	0.53	-0.27	0.65	-0.50	0.25	-1.90	1.40
		TAAM	-0.07	0.13	-0.13	0.26	-0.30	0.25	-1.48	1.36
	4	IAM	-0.18	0.53	-0.27	0.64	-0.45	0.18	X	X
		TAAM	-0.07	0.13	-0.13	0.26	-0.16	0.17	X	X
0.38* Å	1	IAM	-3.70	5.41	-2.37	4.13	-1.75	2.12	Θ	Θ
		TAAM	-3.54	5.68	-2.44	3.90	-1.49	2.35	Θ	Θ
	2	IAM	-0.29	0.54	-0.36	0.79	-1.67	0.39	Θ	Θ
		TAAM	-0.53	0.38	-0.54	0.46	-1.44	0.32	Θ	Θ
	3	IAM	-0.23	0.55	-0.36	0.79	-0.71	0.40	Θ	Θ
		TAAM	-0.14	0.13	-0.24	0.42	-0.33	0.29	Θ	Θ
	4	IAM	-0.23	0.55	-0.36	0.79	-0.53	0.16	Θ	Θ
		TAAM	-0.14	0.13	-0.24	0.42	-0.15	0.17	Θ	Θ
					a,b = 0					
0.83 Å	1	IAM	-0.61	0.76	-0.39	0.61	-0.93	0.87	-1.37	1.36
		TAAM	-0.53	0.62	-0.48	0.43	-0.99	1.21	-1.21	1.37
	2	IAM	-0.44	0.37	-0.23	0.18	X	X	X	X
		TAAM	-0.31	0.27	-0.23	0.24	-0.81	1.10	X	X
	3	IAM	-0.32	0.34	-0.24	0.17	X	X	X	X
		TAAM	-0.11	0.12	-0.11	0.10	-1.36	1.82	X	X
	4	IAM	X	X	–	–	–	–	–	–
		TAAM	-0.09	0.11	–	–	–	–	–	–
0.6 Å	1	IAM	-1.48	1.81	-0.73	1.66	-1.99	1.78	-1.84	1.70
		TAAM	-1.24	1.84	-0.93	1.34	-1.55	1.78	-1.52	1.46
	2	IAM	-0.99	0.77	-0.27	0.67	X	X	X	X
		TAAM	-0.59	0.60	-0.37	0.28	-1.34	1.87	X	X
	3	IAM	-0.80	0.61	-0.27	0.67	X	X	X	X
		TAAM	-0.25	0.25	-0.13	0.26	-2.64	2.04	X	X
	4	IAM	X	X	–	–	–	–	–	–
		TAAM	-0.22	0.25	–	–	–	–	–	–
0.38* Å	1	IAM	-4.88	4.99	-2.21	4.36	-9.71	4.15	Θ	Θ
		TAAM	-4.38	6.13	-2.37	4.07	-2.97	3.79	Θ	Θ
	2	IAM	-2.47	2.18	-0.36	0.79	X	X	Θ	Θ
		TAAM	-1.46	1.42	-0.54	0.49	-2.31	3.77	Θ	Θ
	3	IAM	-2.27	2.15	-0.36	0.79	X	X	Θ	Θ
		TAAM	-0.62	0.59	-0.24	0.43	-4.42	2.74	Θ	Θ
	4	IAM	X	X	-0.36	0.79	–	–	Θ	Θ
		TAAM	-0.52	0.58	-0.24	0.43	–	–	Θ	Θ

Overall scaling factor

Resolution	Option	Model	xSFth	xSFex	x100 eSFth	eSFex	xSFth	xSFex	x100 eSFth	eSFex
			a,b-fitted				a,b = 0			
0.83 Å	1	IAM	1.041	1.093	91.9	3.991	1.032	1.081	86.6	3.637
		TAAM	0.975	1.020	101.6	3.983	0.977	1.021	101.6	3.613
	2	IAM	1.047	1.103	92.1	X	1.028	1.102	X	X
		TAAM	0.975	1.024	102.0	X	0.978	1.024	102.2	X
	3	IAM	1.054	1.108	91.6	X	1.037	1.109	X	X
		TAAM	0.990	1.044	100.6	X	0.994	1.043	100.9	X
	4	IAM	1.057	1.113	91.9	X	X	–	–	–
		TAAM	0.992	1.045	100.9	X	0.996	–	–	–
0.6 Å	1	IAM	1.015	1.045	95.9	4.540	1.029	1.025	87.3	4.070
		TAAM	0.983	1.015	101.0	4.392	0.979	1.011	101.7	4.052
	2	IAM	1.011	1.041	96.5	4.627	1.028	1.026	X	X
		TAAM	0.986	1.017	101.3	4.426	0.980	1.012	102.2	X
	3	IAM	1.013	1.042	96.7	4.633	1.033	1.029	X	X
		TAAM	0.994	1.024	100.5	4.412	0.995	1.025	101.1	X
	4	IAM	1.013	1.042	96.8	X	X	–	–	–
		TAAM	0.994	1.024	100.4	X	0.996	–	–	–
0.38* Å	1	IAM	1.000	1.009	99.2	Θ	1.027	0.987	87.8	Θ
		TAAM	0.984	0.999	99.6	Θ	0.980	0.983	101.7	Θ
	2	IAM	1.003	1.002	101.5	Θ	1.027	1.000	X	Θ
		TAAM	0.995	0.999	100.3	Θ	0.981	0.995	102.1	Θ
	3	IAM	1.004	1.003	99.2	Θ	1.031	1.003	X	Θ
		TAAM	0.999	1.004	100.3	Θ	0.995	1.002	101.1	Θ
	4	IAM	1.004	1.003	99.2	Θ	X	1.003	–	Θ
		TAAM	0.999	1.004	100.1	Θ	0.996	1.002	–	Θ

Weighting scheme parameters

Resolution	Option	Model	xSFth		xSFex		eSFth		eSFex	
			a	b	a	b	a	b	a	b
0.83 Å	1	IAM	0.063	1.798	0.071	1.443	0.100	0.204	0.200	0.000
		TAAM	0.027	1.870	0.034	1.331	0.059	0.120	0.163	4.088
	2	IAM	0.040	0.506	0.047	0.454	0.094	0.117	X	X
		TAAM	0.026	0.440	0.025	0.308	0.073	0.037	X	X
	3	IAM	0.036	0.395	0.046	0.360	0.083	0.019	X	X
		TAAM	0.007	0.025	0.007	0.000	0.082	0.000	X	X
	4	IAM	0.036	0.367	0.047	0.319	0.100	0.019	X	X
		TAAM	0.007	0.015	0.003	0.000	0.082	0.000	X	X
0.6 Å	1	IAM	0.073	1.770	0.092	1.275	0.120	0.069	0.200	0.000
		TAAM	0.179	1.237	0.042	1.343	0.096	0.055	0.190	1.327
	2	IAM	0.060	0.206	0.077	0.213	0.091	0.019	0.200	0.000
		TAAM	0.034	0.136	0.033	0.134	0.071	0.014	0.175	1.220
	3	IAM	0.058	0.143	0.077	0.168	0.096	0.005	0.200	0.000
		TAAM	0.010	0.007	0.000	0.017	0.059	0.000	0.170	1.221
	4	IAM	0.060	0.126	0.077	0.160	0.037	0.000	X	X
		TAAM	0.010	0.007	0.000	0.017	0.047	0.000	X	X
0.38* Å	1	IAM	0.108	1.345	0.129	0.996	0.200	0.000	Θ	Θ
		TAAM	0.017	0.007	0.089	1.073	0.061	0.005	Θ	Θ
	2	IAM	0.056	0.019	0.079	0.047	0.100	0.000	Θ	Θ
		TAAM	0.034	0.012	0.035	0.058	0.059	0.005	Θ	Θ
	3	IAM	0.055	0.008	0.071	0.035	0.044	0.000	Θ	Θ
		TAAM	0.010	0.002	0.016	0.019	0.059	0.005	Θ	Θ
	4	IAM	0.055	0.008	0.076	0.036	0.044	0.000	Θ	Θ
		TAAM	0.010	0.002	0.016	0.019	0.059	0.005	Θ	Θ

Mean e.s.d.s of X-H bonds [Å]

Resolution	Option	Model	xSFth	xSFex	eSFth	eSFex	xSFth	xSFex	eSFth	eSFex
			a,b-fitted				a,b = 0			
0.83 Å	3	IAM	0.013	0.015	0.008	X	0.017	0.014	X	X
		TAAM	0.004	0.006	0.004	X	0.005	0.006	0.020	X
	4	IAM	0.012	0.014	0.007	X	X	–	–	–
		TAAM	0.003	0.006	0.003	X	0.004	–	–	–
0.6 Å	3	IAM	0.010	0.013	0.004	0.043	0.011	0.012	X	X
		TAAM	0.003	0.006	0.002	0.044	0.003	0.007	0.010	X
	4	IAM	0.009	0.012	0.000	X	X	–	–	–
		TAAM	0.002	0.006	0.001	X	0.003	–	–	–
0.38* Å	3	IAM	0.005	0.010	0.002	Θ	0.006	0.009	X	Θ
		TAAM	0.002	0.007	0.002	Θ	0.002	0.007	0.005	Θ
	4	IAM	0.005	0.010	0.001	Θ	X	0.010	–	Θ
		TAAM	0.001	0.007	0.001	Θ	0.002	0.007	–	Θ

RMSD of refined X-H bonds from neutron lengths (for carbamazepine) [Å]

Resolution	Option	Model	xSFth	xSFex	eSFth	eSFex	xSFth	xSFex	eSFth	eSFex
			a,b-fitted				a,b = 0			
0.83 Å	3	IAM	0.102	0.112	0.024	X	0.084	0.111	X	X
		TAAM	0.016	0.020	0.013	X	0.023	0.020	0.073	X
	4	IAM	0.098	0.108	0.026	X	X	–	–	–
		TAAM	0.013	0.018	0.008	X	0.020	–	–	–
0.6 Å	3	IAM	0.095	0.105	0.014	0.078	0.112	0.107	X	X
		TAAM	0.013	0.022	0.007	0.075	0.023	0.025	0.053	X
	4	IAM	0.094	0.106	0.012	X	X	–	–	–
		TAAM	0.012	0.022	0.004	X	0.021	–	–	–
0.38* Å	3	IAM	0.095	0.102	0.013	Θ	0.123	0.110	X	Θ
		TAAM	0.013	0.026	0.005	Θ	0.023	0.025	0.055	Θ
	4	IAM	0.096	0.104	0.008	Θ	X	0.113	–	Θ
		TAAM	0.011	0.027	0.004	Θ	0.020	0.025	–	Θ

ME of refined X-H bonds from neutron lengths (for carbamazepine) [Å]

Resolution	Option	Model	xSFth	xSFex	eSFth	eSFex	xSFth	xSFex	eSFth	eSFex
			a,b-fitted				a,b = 0			
0.83 Å	3	IAM	-0.101	-0.111	0.021	X	-0.078	-0.109	X	X
		TAAM	-0.012	-0.018	0.000	X	-0.016	-0.018	0.019	X
	4	IAM	-0.098	-0.107	0.024	X	X	–	–	–
		TAAM	-0.009	-0.015	0.001	X	-0.010	–	–	–
0.6 Å	3	IAM	-0.094	-0.102	0.011	0.019	-0.106	-0.103	X	X
		TAAM	-0.008	-0.019	-0.001	-0.017	-0.016	-0.017	0.025	X
	4	IAM	-0.093	-0.103	0.006	X	X	–	–	–
		TAAM	-0.007	-0.019	-0.003	X	-0.012	–	–	–
0.38* Å	3	IAM	-0.091	-0.097	0.004	Θ	-0.117	-0.105	X	Θ
		TAAM	-0.007	-0.022	0.000	Θ	-0.015	-0.021	0.027	Θ
	4	IAM	-0.090	-0.099	0.003	Θ	X	-0.108	–	Θ
		TAAM	-0.006	-0.023	0.000	Θ	-0.011	-0.021	–	Θ

Mean of Uiso/Ueq e.s.d.s for non-H atoms [Å^2]						
Resolution	Option	Model	xSFth	xSFex	eSFth	eSFex
				a,b - fitted		
0.83 Å	1	IAM	0.00039	0.00039	0.00049	0.00192
		TAAM	0.00031	0.00030	0.00040	0.00216
	2	IAM	0.00020	0.00028	0.00041	X
		TAAM	0.00018	0.00019	0.00030	X
	3	IAM	0.00020	0.00026	0.00030	X
		TAAM	0.00006	0.00009	0.00020	X
	4	IAM	0.00020	0.00026	0.00029	X
		TAAM	0.00005	0.00009	0.00017	X
0.6 Å	1	IAM	0.00017	0.00017	0.00020	0.00102
		TAAM	0.00017	0.00015	0.00019	0.00116
	2	IAM	0.00007	0.00009	0.00016	0.00111
		TAAM	0.00005	0.00006	0.00013	0.00124
	3	IAM	0.00007	0.00009	0.00009	0.00113
		TAAM	0.00002	0.00004	0.00010	0.00126
	4	IAM	0.00007	0.00009	0.00008	X
		TAAM	0.00001	0.00004	0.00004	X
0.38* Å	1	IAM	0.00007	0.00009	0.00010	Θ
		TAAM	0.00007	0.00008	0.00009	Θ
	2	IAM	0.00001	0.00004	0.00005	Θ
		TAAM	0.00001	0.00003	0.00005	Θ
	3	IAM	0.00001	0.00004	0.00002	Θ
		TAAM	0.00000	0.00002	0.00002	Θ
	4	IAM	0.00001	0.00004	0.00001	Θ
		TAAM	0.00000	0.00002	0.00001	Θ
				a,b = 0		
0.83 Å	1	IAM	0.00060	0.00043	0.00098	0.0012
		TAAM	0.00041	0.00038	0.00099	0.0012
	2	IAM	0.00047	0.00029	X	X
		TAAM	0.00030	0.00020	0.00097	X
	3	IAM	0.00040	0.00027	X	X
		TAAM	0.00011	0.00009	0.0009	X
	4	IAM	X	–	–	–
		TAAM	0.00010	–	–	–
0.6 Å	1	IAM	0.00029	0.00017	0.00055	0.00102
		TAAM	0.00019	0.00016	0.00055	0.00098
	2	IAM	0.00009	0.00009	X	X
		TAAM	0.00014	0.00006	0.00054	X
	3	IAM	0.00019	0.00008	X	X
		TAAM	0.00005	0.00004	0.00045	X
	4	IAM	X	–	–	–
		TAAM	0.00004	–	–	–
0.38* Å	1	IAM	0.00013	0.00009	0.00029	Θ
		TAAM	0.00009	0.00008	0.00029	Θ
	2	IAM	0.00020	0.00004	X	Θ
		TAAM	0.00006	0.00003	0.00028	Θ
	3	IAM	0.00009	0.00003	X	Θ
		TAAM	0.00002	0.00002	0.00020	Θ
	4	IAM	X	0.00003	–	Θ
		TAAM	0.00002	0.00002	–	Θ

Mean of Uiso/Ueq e.s.d.s for H atoms [Å^2]

Resolution	Option	Model	xSFth	xSFex	eSFth	eSFex
				a,b - fitted		
0.83 Å	3	IAM	0.0030	0.0031	0.0017	X
		TAAM	0.0010	0.0014	0.0008	X
	4	IAM	0.0031	0.0033	0.0016	X
		TAAM	0.0008	0.0013	0.0006	X
0.6 Å	3	IAM	0.0020	0.0028	0.0007	0.0082
		TAAM	0.0006	0.0014	0.0004	0.0093
	4	IAM	0.0020	0.0030	0.0004	X
		TAAM	0.0005	0.0014	0.0001	X
0.38 Å	3	IAM	0.0010	0.0020	0.0002	Θ
		TAAM	0.0003	0.0014	0.0002	Θ
	4	IAM	0.0011	0.0023	0.0001	Θ
		TAAM	0.0003	0.0015	0.0002	Θ
				a,b = 0		
0.83 Å	3	IAM	0.0053	0.0030	X	X
		TAAM	0.0016	0.0014	0.0061	X
	4	IAM	X	–	–	–
		TAAM	0.0015	–	–	–
0.6 Å	3	IAM	0.0032	0.0025	X	X
		TAAM	0.0010	0.0014	0.0038	X
	4	IAM	X	–	–	–
		TAAM	0.0009	–	–	–
0.38 Å	3	IAM	0.0017	0.0018	X	Θ
		TAAM	0.0005	0.0014	0.0019	Θ
	4	IAM	X	0.0019	–	Θ
		TAAM	0.0005	0.0014	–	Θ

Average similarity index (non-H) [Å]

Resolution	Option	Model	xSFth	xSFex	eSFth	eSFex	xSFth	xSFex	eSFth	eSFex
				a,b-fitted				a,b=0		
0.83 Å	2	IAM	2.12	5.82	11.08	X	2.86	5.97	X	X
		TAAM	0.82	1.40	0.39	X	1.15	1.54	8.20	X
	3	IAM	2.36	6.14	14.87	X	2.62	6.32	X	X
		TAAM	0.10	2.15	0.22	X	0.18	2.15	5.44	X
	4	IAM	2.55	6.42	10.32	X	X	–	–	–
		TAAM	0.08	2.25	0.28	X	0.17	–	–	–
0.6 Å	2	IAM	0.09	1.49	0.22	7.70	0.92	1.24	X	X
		TAAM	0.05	0.98	0.10	7.68	0.22	0.97	6.46	X
	3	IAM	0.10	1.52	0.26	8.41	0.89	1.29	X	X
		TAAM	0.03	1.12	0.05	7.93	0.06	1.15	4.32	X
	4	IAM	0.10	1.52	0.07	X	X	–	–	–
		TAAM	0.03	1.12	0.07	X	0.06	–	–	–
0.38* Å	2	IAM	0.03	0.72	0.08	Θ	0.56	0.69	X	Θ
		TAAM	0.02	0.64	0.01	Θ	0.10	0.63	5.44	Θ
	3	IAM	0.03	0.73	0.00	Θ	0.53	0.71	X	Θ
		TAAM	0.03	0.69	0.00	Θ	0.04	0.69	3.99	Θ
	4	IAM	0.03	0.73	0.04	Θ	X	0.71	–	Θ
		TAAM	0.03	0.69	0.05	Θ	0.04	0.69	–	Θ

Average similarity index (H-atoms) [Å]

				a,b-fitted			a,b = 0	
Resolution	Option	Model	xSFth	xSFex	eSFth	eSFex	xSFth	xSFex
0.83 Å	4	IAM	17.32	10.16	1.63	X	X	–
		TAAM	1.05	1.71	1.21	X	9.92	–
0.6 Å	4	IAM	7.45	6.65	0.38	X	X	–
		TAAM	1.11	2.41	0.13	X	9.42	–
0.38* Å	4	IAM	11.21	9.08	0.17	Θ	X	7.89
		TAAM	1.25	3.26	0.09	Θ	8.23	3.04

RMSD of Uiso/Ueq for non-H atoms refined from neutron data [Å²]							% of average reference Uiso/Ueq [%]			
Resolution	Option	Model	xSFth	xSFex	eSFth	eSFex	xSFth	xSFex	eSFth	eSFex
						a,b-fitted				
0.83 Å	1	IAM	0.00245	0.00626	0.00413	0.00551	22.1	56.3	37.2	49.6
		TAAM	0.00121	0.00238	0.00117	0.00426	10.9	21.4	10.5	38.3
	2	IAM	0.00296	0.00693	0.00387	X	26.6	62.4	34.8	X
		TAAM	0.00110	0.00271	0.00141	X	9.9	24.4	12.7	X
	3	IAM	0.00331	0.00722	0.00477	X	29.8	65.0	42.9	X
		TAAM	0.00035	0.00362	0.00024	X	3.2	32.6	2.2	X
	4	IAM	0.00352	0.00746	0.00409	X	31.7	67.1	36.8	X
		TAAM	0.00027	0.00371	0.00096	X	2.4	33.4	8.6	X
0.6 Å	1	IAM	0.00045	0.00286	0.00100	0.00459	4.1	25.7	9.0	41.3
		TAAM	0.00041	0.00202	0.00064	0.00366	3.7	18.2	5.8	32.9
	2	IAM	0.00039	0.00283	0.00072	0.00632	3.5	25.5	6.5	56.9
		TAAM	0.00031	0.00216	0.00075	0.00504	2.8	19.4	6.8	45.4
	3	IAM	0.00042	0.00285	0.00098	0.00643	3.8	25.7	8.8	57.9
		TAAM	0.00015	0.00234	0.00027	0.00486	1.4	21.1	2.4	43.7
	4	IAM	0.00042	0.00286	0.00048	X	3.8	25.7	4.3	X
		TAAM	0.00014	0.00235	0.00057	X	1.3	21.2	5.1	X
0.38* Å	1	IAM	0.00028	0.00165	0.00079	Θ	2.5	14.9	7.1	Θ
		TAAM	0.00039	0.00145	0.00022	Θ	3.5	13.1	2.0	Θ
	2	IAM	0.00015	0.00171	0.00066	Θ	1.4	15.4	5.9	Θ
		TAAM	0.00008	0.00159	0.00033	Θ	0.7	14.3	3.0	Θ
	3	IAM	0.00016	0.00173	0.00004	Θ	1.4	15.6	0.4	Θ
		TAAM	0.00009	0.00166	0.00005	Θ	0.8	14.9	0.5	Θ
	4	IAM	0.00016	0.00172	0.00040	Θ	1.4	15.5	3.6	Θ
		TAAM	0.00009	0.00166	0.00046	Θ	0.8	14.9	4.1	Θ
						a,b = 0				
0.83 Å	1	IAM	0.00191	0.00647	0.00860	0.00638	17.2	58.1	77.4	57.4
		TAAM	0.00142	0.00312	0.00263	0.00236	12.8	28.1	23.6	21.2
	2	IAM	0.00189	0.00709	X	X	17.0	63.9	X	X
		TAAM	0.00122	0.00290	0.00316	X	11.0	26.1	28.5	X
	3	IAM	0.00245	0.00739	X	X	22.1	66.5	X	X
		TAAM	0.00018	0.00362	0.00242	X	1.6	32.6	21.8	X
	4	IAM	X	–	–	–	X	–	–	–
		TAAM	0.00018	–	–	–	1.6	–	–	–
0.6 Å	1	IAM	0.00114	0.00294	0.00721	0.00207	10.3	26.4	64.9	18.6
		TAAM	0.00084	0.00252	0.00266	0.00448	7.5	22.7	24.0	40.3
	2	IAM	0.00123	0.00250	X	X	11.1	22.5	X	X
		TAAM	0.00069	0.00213	0.00295	X	6.2	19.2	26.5	X
	3	IAM	0.00141	0.00257	X	X	12.7	23.1	X	X
		TAAM	0.00013	0.00239	0.00207	X	1.2	21.5	18.7	X
	4	IAM	X	–	–	–	X	–	–	–
		TAAM	0.00012	–	–	–	1.1	–	–	–
0.38* Å	1	IAM	0.00080	0.00173	0.00652	Θ	7.2	15.5	58.7	Θ
		TAAM	0.00069	0.00162	0.00267	Θ	6.2	14.6	24.0	Θ
	2	IAM	0.00099	0.00168	X	Θ	8.9	15.1	X	Θ
		TAAM	0.00049	0.00156	0.00291	Θ	2.2	14.0	26.2	Θ
	3	IAM	0.00110	0.00171	X	Θ	9.9	15.4	X	Θ
		TAAM	0.00010	0.00166	0.00210	Θ	0.9	15.0	18.9	Θ
	4	IAM	X	0.00171	–	Θ	X	15.4	–	Θ
		TAAM	0.00010	0.00166	–	Θ	0.9	15.0	–	Θ

RMSD of Uiso/Ueq for H-atoms refined from neutron data [Å²]							% of average reference Uiso/Ueq [%]			
Resolution	Option	Model	xSFth	xSFex	eSFth	eSFex	xSFth	xSFex	eSFth	eSFex
						a,b - fitted				
0.83 Å	3	IAM	0.0122	0.0083	0.0024	X	41.5	28.4	8.0	X
		TAAM	0.0030	0.0025	0.0019	X	10.2	8.3	6.5	X
	4	IAM	0.0111	0.0067	0.0021	X	37.8	23.0	7.2	X
		TAAM	0.0028	0.0029	0.0016	X	9.7	9.9	5.5	X
0.6 Å	3	IAM	0.0084	0.0060	0.0008	0.0187	28.5	20.5	2.7	63.5
		TAAM	0.0028	0.0020	0.0015	0.0161	9.7	6.8	5.2	54.6
	4	IAM	0.0074	0.0050	0.0011	X	25.1	17.0	3.6	X
		TAAM	0.0027	0.0023	0.0004	X	9.1	7.8	1.5	X
0.38 Å	3	IAM	0.0069	0.0056	0.0011	Θ	23.6	19.1	3.8	Θ
		TAAM	0.0028	0.0017	0.0011	Θ	9.6	5.8	3.8	Θ
	4	IAM	0.0053	0.0047	0.0010	Θ	18.2	16.0	3.5	Θ
		TAAM	0.0026	0.0020	0.0003	Θ	8.8	6.7	0.9	Θ
						a,b = 0				
0.83 Å	3	IAM	0.0150	0.0083	X	X	51.1	28.2	X	X
		TAAM	0.0064	0.0024	0.0074	X	21.9	8.3	25.2	X
	4	IAM	X	–	–	–	X	–	–	–
		TAAM	0.0052	–	–	–	17.6	–	–	–
0.6 Å	3	IAM	0.0174	0.0067	X	X	59.0	22.9	X	X
		TAAM	0.0061	0.0020	0.0080	X	20.7	6.7	27.2	X
	4	IAM	X	–	–	–	X	–	–	–
		TAAM	0.0052	–	–	–	17.8	–	–	–
0.38 Å	3	IAM	0.0188	0.0065	X	Θ	63.8	22.2	X	Θ
		TAAM	0.0060	0.0017	0.0074	Θ	20.5	5.6	25.0	Θ
	4	IAM	X	0.0050	–	Θ	X	17.0	–	Θ
		TAAM	0.0053	0.0020	–	Θ	17.9	6.7	–	Θ

ME of Uiso/Ueq for non-H atoms refined from neutron data [Å^2]

Resolution	Option	Model	xSFth	xSFex	eSFth	eSFex
a,b - fitted						
0.83 Å	1	IAM	0.00244	0.00624	-0.00410	-0.00478
		TAAM	-0.00119	0.00236	0.00112	-0.00333
	2	IAM	0.00295	0.00692	-0.00383	X
		TAAM	-0.00109	0.00269	0.00138	X
	3	IAM	0.00331	0.00721	-0.00477	X
		TAAM	-0.00031	0.00361	0.00020	X
	4	IAM	0.00351	0.00745	-0.00407	X
		TAAM	-0.00022	0.00370	0.00093	X
0.6 Å	1	IAM	0.00043	0.00285	-0.00096	0.00404
		TAAM	-0.00035	0.00200	0.00059	0.00319
	2	IAM	0.00038	0.00282	-0.00065	0.00589
		TAAM	-0.00029	0.00214	0.00070	0.00469
	3	IAM	0.00041	0.00284	-0.00097	0.00597
		TAAM	-0.00012	0.00233	0.00023	0.00449
	4	IAM	0.00041	0.00285	-0.00042	X
		TAAM	-0.00010	0.00233	0.00054	X
0.38* Å	1	IAM	-0.00006	0.00162	0.00071	Θ
		TAAM	-0.00025	0.00141	0.00000	Θ
	2	IAM	0.00013	0.00169	0.00064	Θ
		TAAM	0.00001	0.00157	0.00027	Θ
	3	IAM	0.00014	0.00171	0.00000	Θ
		TAAM	0.00005	0.00164	0.00003	Θ
	4	IAM	0.00014	0.00171	0.00038	Θ
		TAAM	0.00005	0.00164	0.00043	Θ
a,b = 0						
0.83 Å	1	IAM	0.00179	0.00646	-0.00853	-0.00599
		TAAM	-0.00130	0.00311	0.00162	-0.00055
	2	IAM	0.00167	0.00708	X	X
		TAAM	-0.00110	0.00288	0.00251	X
	3	IAM	0.00234	0.00738	X	X
		TAAM	-0.00005	0.00361	0.00138	X
	4	IAM	X	–	–	–
		TAAM	0.00001	–	–	–
0.6 Å	1	IAM	0.00105	0.00292	-0.00713	0.00149
		TAAM	-0.00077	0.00250	0.00175	0.00421
	2	IAM	0.00116	0.00249	X	X
		TAAM	-0.00064	0.00211	0.00232	X
	3	IAM	0.00137	0.00255	X	X
		TAAM	-0.00007	0.00237	0.00109	X
	4	IAM	X	–	–	–
		TAAM	-0.00005	–	–	–
0.38* Å	1	IAM	0.00074	0.00170	-0.00642	Θ
		TAAM	-0.00064	0.00159	0.00176	Θ
	2	IAM	0.00095	0.00168	X	Θ
		TAAM	-0.00045	0.00166	0.00228	Θ
	3	IAM	0.00106	0.00171	X	Θ
		TAAM	-0.00004	0.00169	0.00116	Θ
	4	IAM	X	0.00169	–	Θ
		TAAM	-0.00002	0.00164	–	Θ

ME of Uiso/Ueq for H-atoms refined from neutron data [Å^2]						
Resolution	Option	Model	xSFth	xSFex	eSFth	eSFex
				a,b - fitted		
0.83 Å	3	IAM	-0.0112	-0.0073	0.0017	X
		TAAM	-0.0026	0.0019	0.0013	X
	4	IAM	-0.0098	-0.0056	0.0016	X
		TAAM	-0.0024	0.0024	-0.0008	X
0.6 Å	3	IAM	-0.0076	-0.0048	0.0005	0.0054
		TAAM	-0.0024	0.0013	-0.0004	0.0039
	4	IAM	-0.0062	-0.0029	0.0007	X
		TAAM	-0.0022	0.0017	0.0000	X
0.38 Å	3	IAM	-0.0056	-0.0043	-0.0009	Θ
		TAAM	-0.0023	0.0006	-0.0010	Θ
	4	IAM	-0.0033	-0.0021	0.0008	Θ
		TAAM	-0.0020	0.0012	0.0002	Θ
				a,b = 0		
0.83 Å	3	IAM	-0.0118	-0.0073	X	X
		TAAM	-0.0040	0.0019	0.0016	X
	4	IAM	X	–	–	–
		TAAM	-0.0030	–	–	–
0.6 Å	3	IAM	-0.0147	-0.0060	X	X
		TAAM	-0.0037	0.0014	0.0023	X
	4	IAM	X	–	–	–
		TAAM	-0.0029	–	–	–
0.38 Å	3	IAM	-0.0166	-0.0059	X	Θ
		TAAM	-0.0035	0.0006	0.0020	Θ
	4	IAM	X	-0.0040	–	Θ
		TAAM	-0.0027	0.0013	–	Θ
